# Combined Effects of Marine Heatwaves and Light Intensity on the Physiological, Transcriptomic, and Metabolomic Profiles of *Undaria pinnatifida*

**DOI:** 10.3390/plants14101419

**Published:** 2025-05-09

**Authors:** Hanmo Song, Yan Liu, Qingli Gong, Xu Gao

**Affiliations:** Key Laboratory of Mariculture, Ministry of Education, Fisheries College, Ocean University of China, Qingdao 266003, China; songhanmo@stu.ouc.edu.cn (H.S.); qingli@vip.sina.com (Q.G.)

**Keywords:** high light stress, marine heatwaves, metabolomics, physiological response, transcriptomics, *Undaria pinnatifida*

## Abstract

Marine heatwaves (MHWs) are spreading across global oceanic regions with unprecedented intensity, frequency, and duration, and are often accompanied by changes in underwater light, thereby imposing multiple stressors on coastal macroalgae. In this study, the effects of MHW intensities (moderate: +3 °C; severe: +6 °C) and light intensities (normal: 90 μmol photons m^−2^ s^−1^; high: 270 μmol photons m^−2^ s^−1^) on cultivated *Undaria pinnatifida* were investigated through an integrated analysis of physiological, transcriptomic, and metabolomic responses. Under moderate MHW conditions, *U. pinnatifida* exhibited enhanced growth and photosynthetic performance, with increased pigment content, improved electron transport, and the early activation of antioxidant defenses. Following severe MHW exposure, the partial recovery of some physiological traits was observed, while photosynthetic capacity, membrane integrity, and energy metabolism remained impaired, and oxidative damage was not fully resolved. High light stress further aggravated stress responses under both MHW intensities by disrupting photoprotection and weakening antioxidant defense systems. These results suggest that *U. pinnatifida* exhibits adaptive capacity under moderate MHWs and delayed physiological damage and incomplete recovery under severe MHWs. High light stress further exacerbates both responses, ultimately affecting yield and quality.

## 1. Introduction

Marine heatwaves (MHWs) have emerged as a major threat to the structural and functional integrity of marine ecosystems under accelerating global climate change. MHWs are defined as extreme warming events during which sea surface temperatures exceed the local seasonal threshold (typically the 90th percentile based on a 30-year historical climatology) for at least five consecutive days, and they are ecologically distinct from the gradual process of long-term ocean warming due to their abrupt onset, short duration, and intensity [1,2]. Long-term observational records reveal that MHWs have intensified in scale and impact over recent decades, exhibiting substantial spatial variability. These trends are projected to intensify over the coming decades, particularly in mid- to high-latitude regions [3,4]. The increasing recurrence of MHWs is disrupting previously stable ecological processes, impairing growth and reproduction in marine organisms, destabilizing trophic interactions, and potentially driving irreversible shifts in community structure [5]. These impacts reduce ecosystem resilience and intensify the survival pressure on thermally sensitive species, highlighting the urgent need to clarify their response mechanisms and adaptive strategies, and to provide an empirical basis for understanding the broader ecological consequences of MHWs [6].

Kelp species contribute fundamentally to the functioning of coastal marine ecosystems by supporting biodiversity, maintaining high levels of primary productivity, and facilitating energy and nutrient transfer across trophic levels [7]. Under the increasing prevalence of MHWs, their ecological functioning has been progressively undermined [8]. In many regions, widespread declines or even local extinctions of kelp forests have been reported, often accompanied by reductions in structural complexity, biodiversity, and ecosystem functionality [9,10]. For instance, the severe MHW event that occurred off the southwestern coast of Australia in 2011 led to the large-scale degradation of *Ecklonia radiata* (Phaeophyceae) forests, where diverse, architecturally complex assemblages were replaced by low-diversity turf beds. This shift significantly reduced primary productivity and carbon sequestration potential, thereby affecting the abundance and distribution of associated faunal communities, including fish, invertebrates, and marine mammals, and ultimately threatening the sustainability of coastal fisheries [11,12]. In certain ecosystems, extreme MHWs have triggered blooms of subcanopy or benthic turf-forming algae, concurrent with significant declines in sessile invertebrate populations. These shifts have restructured trophic linkages and interspecies interactions, facilitating the establishment of thermophilic or non-native species such as *Sargassum* spp., *Lobophora* spp., and *Padina* spp., and potentially contributing to changes in community composition and ecological function, including alterations in energy flow and the simplification of habitat complexity [13,14]. By altering species composition and ecological functions, the loss of kelp forests may weaken the capacity of coastal ecosystems to buffer environmental variability and contribute to broader-scale climatic instabilities [15]. Despite widespread documentation of these ecological impacts of MHW events on kelp species, little is known regarding their self-regulatory mechanisms [16].

During MHW events, reduced cloud cover allows increased solar radiation to penetrate the water column, while stratification induced by elevated temperatures suppresses phytoplankton growth, jointly enhancing the light penetration depth and expanding the vertical zone suitable for photosynthesis [17]. As a result, macroalgae are increasingly exposed to novel light–temperature combinations that differ markedly from historical baselines [18,19]. Under such compound stress conditions, light serves as a critical modulating factor in shaping macroalgal physiological responses to elevated temperatures [20,21]. Excessive radiation may exacerbate heat-induced metabolic disruption by triggering photoinhibition and oxidative stress, while moderate light enhancement may, under certain conditions, support sustained carbon fixation and energy supply, thereby contributing to short-term physiological homeostasis [22,23]. The direction and magnitude of these effects depend on species-specific traits, physiological status, and environmental context [24]. Due to their sessile nature, macroalgae lack behavioral escape mechanisms and rely primarily on internal physiological and molecular regulation to cope with these dynamic stressors [25]. Compared to single-factor MHW stress, the addition of fluctuating light introduces greater environmental complexity, with potentially nonlinear outcomes that remain poorly understood [26,27]. Elucidating the interactive effects of light and temperature is therefore essential for assessing the sensitivity and adaptive capacity of macroalgae under future ocean conditions, particularly increased frequency and intensity of MHWs and variability in light availability.

*Undaria pinnatifida*, a temperate-to-cold-temperate brown macroalga, is widely distributed in coastal East Asia where it maintains stable native populations. This species has successfully spread to numerous coastlines across different sea areas, becoming one of the most prominent invasive macroalgae globally [28,29]. As a representative kelp, *U. pinnatifida* plays a crucial ecological role in fixing photosynthetic carbon, sustaining primary productivity, and driving nearshore nutrient cycling. It also holds significant economic value and is currently one of the most extensively cultivated seaweed species worldwide [30]. In recent years, both natural and aquaculture populations of *U. pinnatifida* have been subjected to increasingly complex environmental stressors. Beyond the long-term trend of ocean warming, marine environments are also experiencing growing fluctuations, including seasonal salinity anomalies, intensified eutrophication, pollutant accumulation, and violent physical disturbances [31,32,33,34]. These stressors may interact to impair photosynthetic capacity, energy metabolism, membrane stability, and reproductive performance, potentially placing *U. pinnatifida* in a more vulnerable physiological state when facing additional extreme events [35]. More critically, certain environmental factors may compromise its stress tolerance or disrupt its metabolic balance, thereby increasing susceptibility to other stressors and leading to a highly variable response pattern, often manifested as non-linear or unpredictable physiological reactions. Given the inevitable exposure of *U. pinnatifida* to MHWs and concurrent changes in light conditions in both natural habitats and farming areas, clarifying its response patterns and underlying regulatory mechanisms is imperative [36].

This study investigated the effects of MHW intensity and light intensity on the growth, photosynthetic performance, antioxidant responses, and transcriptomic and metabolomic profiles of cultivated *U. pinnatifida*, in order to elucidate its regulatory mechanisms under their combined stress. These findings are expected to advance our understanding of the adaptive strategies of kelp species in response to MHW-induced environmental turbulence and provide a scientific basis for the risk assessment and management of kelp aquaculture under future climate change scenarios, such as adjusting cultivation depth to avoid excessive light exposure and breeding for resilience.

## 2. Results

### 2.1. Relative Growth Rate

**Figure 1:**
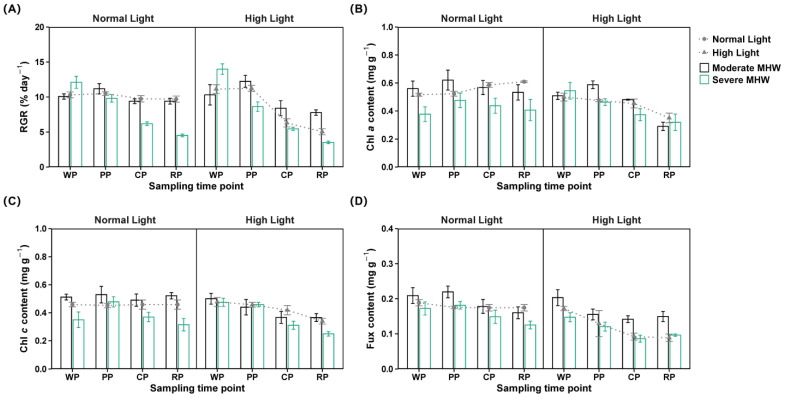
Growth and pigment contents of *Undaria pinnatifida* under different marine heatwave and light treatments. (**A**) Relative growth rate, (**B**) chlorophyll *a* content, (**C**) chlorophyll *c* content, and (**D**) fucoxanthin content. WP, PP, CP, and RP represent the sampling time points at the end of the warming, peak, cooling, and recovery phases, respectively. Data represent mean ± SD (n = 3).

Three-way repeated measures ANOVA revealed significant interactions among MHW intensity, light intensity, and sampling time for the RGR, with a large effect size (Figure 1A; Table S2). During the warming phase, the RGR of the severe MHW under normal light (S_MHW_N_Light_) group was significantly higher than in the moderate MHW under normal light (M_MHW_N_Light_) group. In contrast, during the cooling and recovery phases, S_MHW_N_Light_ exhibited significantly lower RGR values than both M_MHW_N_Light_ and the control (Ctrl). During these phases, the moderate MHW under high light (M_MHW_H_Light_) group displayed a significantly higher RGR than the severe MHW under high light (S_MHW_H_Light_) group and the high light only (H_Light_) group (Table S3). The RGR of M_MHW_N_Light_ and S_MHW_N_Light_ increased during the peak phase, decreased during the cooling phase, and stabilized at similar levels during the recovery phase. M_MHW_H_Light_ and S_MHW_H_Light_ showed a gradual, significant decline in the RGR throughout the experimental period (Table S5).

### 2.2. Pigment Content

Three-way repeated measures ANOVA revealed significant interactions among MHW intensity, light intensity, and sampling time for the Fux content, with a large effect size. For Chl *a* and Chl *c*, no three-way interaction was detected, though a significant two-way interaction between MHW intensity and light intensity was observed, with a large effect size (Table S2).

Under normal light conditions, the Chl *a* content in both the moderate and severe MHW groups was significantly higher than that in the Ctrl group (Figure 1B; Table S6).

The Chl *c* content in the moderate MHW group was significantly higher than in both the severe MHW and Ctrl groups, while the severe MHW group exhibited significantly lower Chl *c* content values than the Ctrl group (Figure 1C; Table S6).

During the peak phase, the Fux content in M_MHW_N_Light_ was significantly higher than that in the Ctrl (Figure 1D; Table S3). During the peak and cooling phases, the Fux content in M_MHW_N_Light_ exceeded that in M_MHW_H_Light_ (Table S4). The Fux content in M_MHW_N_Light_ showed a gradual decline, whereas those in M_MHW_H_Light_, S_MHW_N_Light_, S_MHW_H_Light_, and H_Light_ showed significant reductions during recovery (Table S5).

### 2.3. F_v_/F_m_ and qP

Significant three-way interactions among MHW intensity, light intensity, and sampling time were observed for F_v_/F_m_, with a large effect size. For qP, significant two-way interactions were detected between MHW intensity and light intensity, with a large effect size (Table S2).

**Figure 2:**
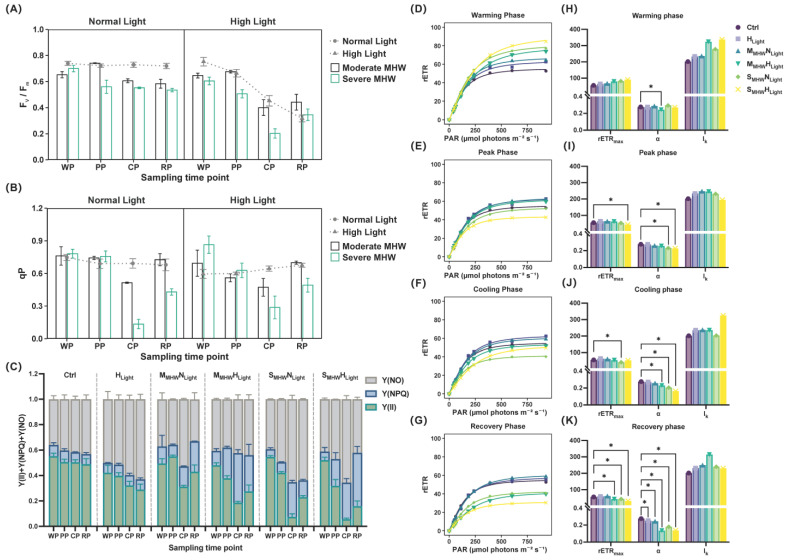
Chlorophyll fluorescence parameters of *Undaria pinnatifida* under different marine heatwave and light treatments. (**A**) F_v_/F_m_; (**B**) qP; (**C**) Y(II), Y(NPQ), and Y(NO), represented as green, blue, and gray stacked areas, respectively; (**D**–**G**) rapid light–response curves, and (**H**–**K**) fitted photosynthetic parameters (rETR_max_, α, and I_k_) during each experimental phase. WP, PP, CP, and RP represent the sampling time points at the end of the warming, peak, cooling, and recovery phases, respectively. Data represent mean ± SD (n = 3). An asterisk (*) denotes statistically significant differences at *p* < 0.05.

At the peak phase, F_v_/F_m_ of S_MHW_N_Light_ was significantly lower than that of M_MHW_N_Light_ and the Ctrl (Figure 2A; Table S3). F_v_/F_m_ of M_MHW_N_Light_ increased at peak, declined during cooling, and remained stable during recovery. F_v_/F_m_ of S_MHW_N_Light_ declined at peak and remained unchanged through cooling and recovery (Table S5).

Under normal light, the qP of the severe MHW group was significantly lower than that of the moderate MHW and Ctrl groups (Figure 2B; Table S6).

### 2.4. PSII Energy Distribution Parameters

Significant three-way interactions among MHW intensity, light intensity, and sampling time were observed for Y(II), Y(NPQ), and Y(NO), with a large effect size (Figure 2C; Table S2).

Y(II) of M_MHW_N_Light_ and S_MHW_N_Light_ was significantly higher than that of the Ctrl group from peak to recovery (Table S3). In both the moderate and severe MHW groups, Y(II) decreased during cooling and increased during recovery (Table S5).

Y(NPQ) was significantly lower in M_MHW_N_Light_ than in S_MHW_N_Light_ during cooling and recovery (Table S3), and lower in S_MHW_N_Light_ than in S_MHW_H_Light_ during peak and cooling phases (Table S4). Y(NPQ) of S_MHW_N_Light_ increased during cooling and recovery, while that of S_MHW_H_Light_ showed a continuous increase (Table S5).

Y(NO) of M_MHW_N_Light_ was significantly lower than that in the Ctrl group from peak to recovery (Table S3) and was also lower than that in M_MHW_H_Light_ (Table S4). Y(NO) of S_MHW_N_Light_ increased during the cooling phase (Table S5).

### 2.5. Light–Response Curves and Fitted Parameters

During the warming phase, rETR increased rapidly under low PAR and then plateaued, with all treatment groups showing higher values than the Ctrl (Figure 2D). At the peak phase, differences among treatments narrowed, and rETR in the severe MHW group fell below that of the Ctrl (Figure 2E). In the cooling phase, rETR remained stable, with M_MHW_H_Light_ showing lower rETR values than the Ctrl (Figure 2F). During recovery, rETR declined overall; only rETR of H_Light_ and M_MHW_N_Light_ remained slightly above that of the Ctrl, while for other groups, it was lower (Figure 2G).

Bootstrap resampling was used to analyze the fitted parameters, with significant differences from the control (*p* < 0.05) indicated. At the warming phase, the α in M_MHW_H_Light_ was significantly lower than that in the Ctrl (Figure 2H). At the peak phase, rETR_max_ of S_MHW_H_Light_ was significantly lower than that of the Ctrl, and α was significantly lower in both S_MHW_N_Light_ and S_MHW_H_Light_ (Figure 2I). In the cooling phase, rETR_max_ of S_MHW_N_Light_ was significantly reduced; α was also significantly lower in M_MHW_H_Light_, S_MHW_N_Light_, and S_MHW_H_Light_ (Figure 2J). At the recovery phase, rETR_max_ of M_MHW_H_Light_, S_MHW_N_Light_, and S_MHW_H_Light_ remained significantly below that of the Ctrl; α was significantly lower in all treatment groups (Figure 2K).

### 2.6. Physiological Stress Indicators

Significant three-way interactions among MHW intensity, light intensity, and sampling time were detected for the proline and MDA content, with a large effect size (Table S2).

**Figure 3:**
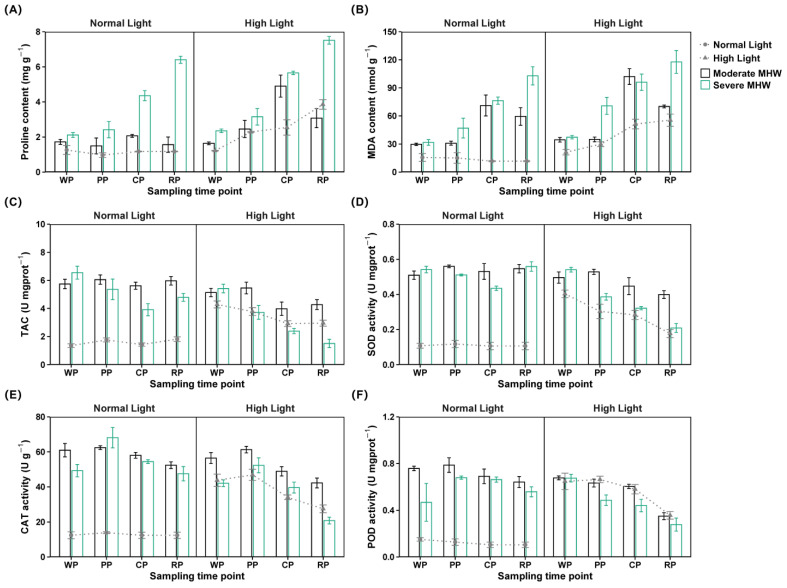
Oxidative stress and antioxidant responses of *Undaria pinnatifida* under different marine heatwave and light treatments: (**A**,**B**) MDA and proline contents and (**C**–**F**) activities of antioxidant enzymes including SOD, TAC, CAT, and POD. WP, PP, CP, and RP represent the sampling time points at the end of the warming, peak, cooling, and recovery phases, respectively. Data represent mean ± SD (n = 3).

From warming to recovery, the proline content in S_MHW_N_Light_ remained significantly higher than that in M_MHW_N_Light_ and the Ctrl (Figure 3A; Table S3). From peak to recovery, the proline content of M_MHW_N_Light_ was significantly lower than that of M_MHW_H_Light_, and the proline content of S_MHW_N_Light_ was lower than that of S_MHW_H_Light_ (Table S4). The proline content of M_MHW_H_Light_ peaked in cooling and declined in recovery, whereas that of S_MHW_N_Light_ and S_MHW_H_Light_ increased from cooling to recovery (Table S5).

The MDA content of M_MHW_N_Light_ and S_MHW_N_Light_ was significantly higher than that of the Ctrl across all MHW phases (Figure 3B). At peak and recovery, the MDA content of S_MHW_H_Light_ was higher than that of M_MHW_H_Light_ and H_Light_ (Table S3). The MDA content of S_MHW_N_Light_ was consistently lower than that of S_MHW_H_Light_ (Table S4). The MDA content of M_MHW_N_Light_ and M_MHW_H_Light_ increased during cooling, whereas that of S_MHW_N_Light_ and S_MHW_H_Light_ increased from warming to recovery (Table S5).

### 2.7. Antioxidant Enzyme Activities

Significant three-way interactions among MHW intensity, light intensity, and sampling time were detected for the TAC and the enzymatic activities of SOD, CAT, and POD, with a large effect size (Table S2).

The TAC of M_MHW_N_Light_ and S_MHW_N_Light_ remained elevated compared to that of the Ctrl (Figure 3C; Table S3). From warming to recovery, the TAC of S_MHW_N_Light_ was higher than that of S_MHW_H_Light_ (Table S4). The TAC of M_MHW_H_Light_ declined during cooling and recovery; that of S_MHW_N_Light_ decreased during peak and cooling but increased in recovery; and that of S_MHW_H_Light_ gradually declined across all phases (Table S5).

The SOD activity of M_MHW_N_Light_ and S_MHW_N_Light_ was significantly higher than that of the Ctrl (Figure 3D; Table S3). During cooling and recovery, the SOD activity of M_MHW_N_Light_ and S_MHW_N_Light_ remained higher than that of their respective high light counterparts (Table S4). The SOD activity of M_MHW_N_Light_ peaked at the peak phase; that of M_MHW_H_Light_ declined during cooling and recovery; and that of S_MHW_N_Light_, S_MHW_H_Light_, and H_Light_ decreased progressively (Table S5).

The CAT activity of M_MHW_N_Light_ remained higher than that of the Ctrl (Figure 3E). From warming to recovery, the CAT activity of M_MHW_H_Light_ was higher than that of S_MHW_H_Light_ and H_Light_ (Table S3). The CAT activity of S_MHW_N_Light_ was higher than that of S_MHW_H_Light_ throughout the same period (Table S4); that of M_MHW_N_Light_ decreased during recovery; and that of the other groups declined gradually from warming to recovery (Table S5).

The POD activity of M_MHW_N_Light_ and S_MHW_N_Light_ remained consistently above that of the Ctrl (Figure 3F; Table S3). From peak to recovery, the POD activity of M_MHW_N_Light_ and S_MHW_N_Light_ was higher than that of M_MHW_H_Light_ and S_MHW_H_Light_ (Table S4). The POD activity of M_MHW_N_Light_ declined in cooling and recovery, whereas that of S_MHW_N_Light_ increased during peak and cooling (Table S5).

### 2.8. Transcriptomic Analysis

#### 2.8.1. Transcriptome Assembly and Annotation

Transcriptomic analysis was conducted on *U. pinnatifida* samples collected at the end of the recovery phase. After quality filtering, approximately 392 million clean reads were obtained, with a sequencing error rate below 0.01%, Q20 and Q30 values exceeding 97% and 93%, respectively, and GC content variation within 2%, indicating high data quality (Table S7). Functional annotations for unigenes were performed using seven public databases (Table S8).

#### 2.8.2. Differential Gene Expression Analysis

Pairwise comparisons were conducted between each treatment group and the control group, including high light only (H_Light__Ctrl), moderate MHW under normal light (M_MHW_N_Light__Ctrl), moderate MHW under high light (M_MHW_H_Light__Ctrl), severe MHW under normal light (S_MHW_N_Light__Ctrl), and severe MHW under high light (S_MHW_H_Light__Ctrl). The number of DEGs identified in each comparison was 7929 (4294 upregulated and 3635 downregulated) in H_Light__Ctrl, 15,668 (8547 upregulated and 7121 downregulated) in M_MHW_N_Light__Ctrl, 12,934 (6792 upregulated and 6142 downregulated) in M_MHW_H_Light__Ctrl, 13,854 (7658 upregulated and 6196 downregulated) in S_MHW_N_Light__Ctrl, and 12,828 (7160 upregulated and 5668 downregulated) in S_MHW_H_Light__Ctrl (Figure S1A). Among these, M_MHW_N_Light__Ctrl contained the highest number of unique DEGs, totaling 3911 (Figure S1B). Hierarchical clustering revealed that moderate and severe MHW groups formed distinct expression clusters, both of which differed from the control and high light groups (Figure S1C).

#### 2.8.3. GO Enrichment Analysis of Differentially Expressed Genes

GO enrichment analysis was performed on DEGs from the five comparison groups (Figure S2). Oxidoreductase activity, catalytic activity, and transmembrane transport were significantly enriched in all comparison groups. Transporter activity and GTPase activity were significantly enriched in the moderate MHW comparison groups. Antioxidant activity, molecular transducer activity, carbohydrate derivative metabolic process, nucleobase-containing small-molecule metabolic process, and catalytic activity acting on a protein were significantly enriched in the severe MHW comparison groups.

#### 2.8.4. KEGG Enrichment Analysis of Differentially Expressed Genes

**Figure 4:**
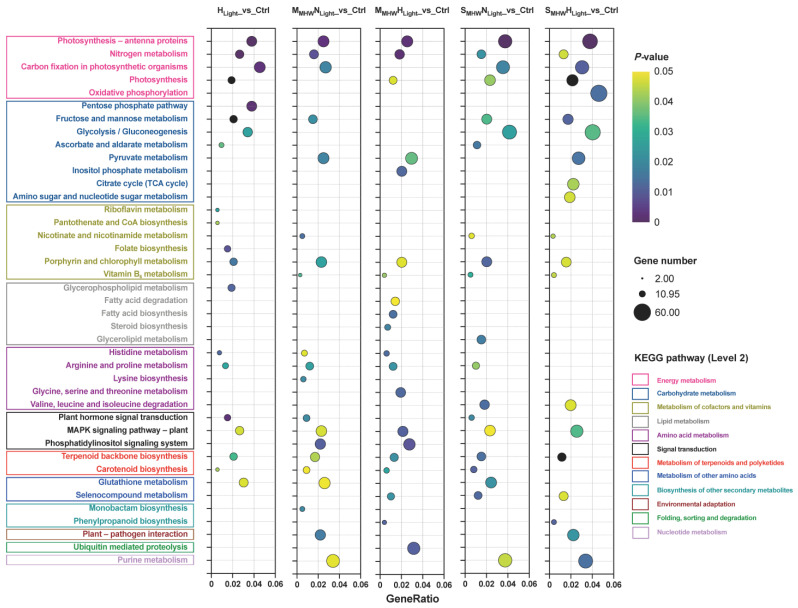
KEGG enrichment analysis of differentially expressed genes in *Undaria pinnatifida* under different marine heatwave and light treatments. Colored boxes represent KEGG secondary pathway classifications. GeneRatio indicates the ratio of target genes to background genes. Circle size represents gene count, and color indicates *p* value (lighter for higher *p* values, darker for lower *p* values).

KEGG pathway enrichment analysis was conducted for DEGs from the five comparison groups, and the top 20 significantly enriched results for each comparison group are presented (Figure 4). Photosynthesis-antenna proteins, nitrogen metabolism, terpenoid backbone biosynthesis, MAPK signaling pathway-plant, and porphyrin and chlorophyll metabolism pathways were significantly enriched in all five comparison groups. The moderate MHW comparison groups commonly showed significant enrichment in carotenoid biosynthesis, pyruvate metabolism, histidine metabolism, and arginine and proline metabolism pathways. The severe MHW comparison groups commonly exhibited significant enrichment in carbon fixation in photosynthetic organisms, glycolysis/gluconeogenesis, fructose and mannose metabolism, purine metabolism, nicotinate and nicotinamide metabolism, selenocompound metabolism, and valine, leucine and isoleucine degradation pathways.

### 2.9. Validation of Representative Gene Expression Patterns Using qRT-PCR

Representative genes associated with photosynthesis (*Lhca1*, *Lhca4*, *PsbA*, *PsbO*, *PsbU*, *Psb27*), antioxidant defense (*SOD1*), and heat shock response (*HSP40*, *HSP70*, *HSP90*) were selected for validation. Their expression trends were in good agreement with the RNA-seq data (Figure S3).

### 2.10. Metabolomic Analysis

#### 2.10.1. Data Quality Assessment and Multivariate Analysis

Untargeted metabolomic profiling was conducted on samples collected during the recovery phase using LC-MS. Both positive and negative ion modes were employed, with datasets merged for subsequent analysis. PCA revealed that the first two components explained 37.5% and 14.9% of the variance, respectively (Figure S4A). PLS-DA further distinguished all treatment groups from the control, confirming robust inter-group separation (Figure S4B). High R^2^ and Q^2^ values validated model quality and predictive capability (Figure S4C). The number of DEMs identified in each comparison was 395 (227 upregulated and 168 downregulated) in H_Light__Ctrl, 552 (204 upregulated and 348 downregulated) in M_MHW_N_Light__Ctrl, 585 (159 upregulated and 426 downregulated) in M_MHW_H_Light__Ctrl, 427 (228 upregulated and 199 downregulated) in S_MHW_N_Light__Ctrl, and 508 (204 upregulated and 304 downregulated) in S_MHW_H_Light__Ctrl (Figure S4D).

#### 2.10.2. KEGG Pathway Analysis of Differentially Expressed Metabolites

**Figure 5:**
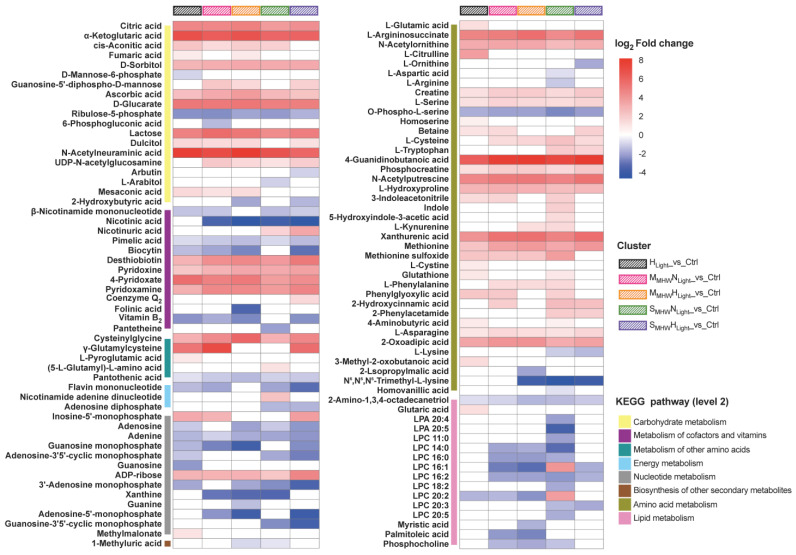
KEGG enrichment analysis and expression profiles of differentially expressed metabolites in *Undaria pinnatifida* under different marine heatwave and light treatments. The color scale represents log_2_ fold change, with red indicating upregulation and blue indicating downregulation. Colored bars denote KEGG secondary pathway annotations of metabolites, while striped color blocks indicate different treatment groups.

KEGG analysis of DEMs from the five comparison groups revealed that 110 DEMs were annotated to KEGG pathways (Figure 5). In all comparison groups, within the carbohydrate metabolism pathway, citric acid, α-ketoglutaric acid, D-sorbitol, ascorbic acid, D-glucarate, lactose, and N-acetylneuraminic acid were significantly upregulated, while ribulose-5-phosphate was significantly downregulated. In the metabolism of cofactors and vitamins pathway, pimelic acid was significantly downregulated, while desthiobiotin, pyridoxine, 4-pyridoxate, and pyridoxamine were significantly upregulated. Under high light conditions, L-(-)-arabitol was significantly upregulated, while biocytin, vitamin B_2_, adenosine, guanosine monophosphate (GMP), and 3′-adenosine monophosphate (3′-AMP) were significantly downregulated. Under severe MHW conditions, L-cysteine and L-tryptophan were significantly upregulated, whereas β-nicotinamide mononucleotide (NMN), nicotinic acid, adenosine, and 3′-AMP were significantly downregulated. In the S_MHW_N_Light__vs_Ctrl group, L-aspartic acid, L-arginine, 20:4-lysophosphatidic acid (LPA), and 20:5-LPA were uniquely enriched and significantly downregulated, whereas indole and 5-hydroxyindole-3-acetic acid (5-HIAA) were significantly upregulated. γ-Glutamylcysteine (γ-Glu-Cys) was significantly upregulated in both the H_Light__vs_Ctrl and S_MHW_H_Light__vs_Ctrl groups.

### 2.11. Integrated KEGG Enrichment of Transcriptome and Metabolome

**Figure 6:**
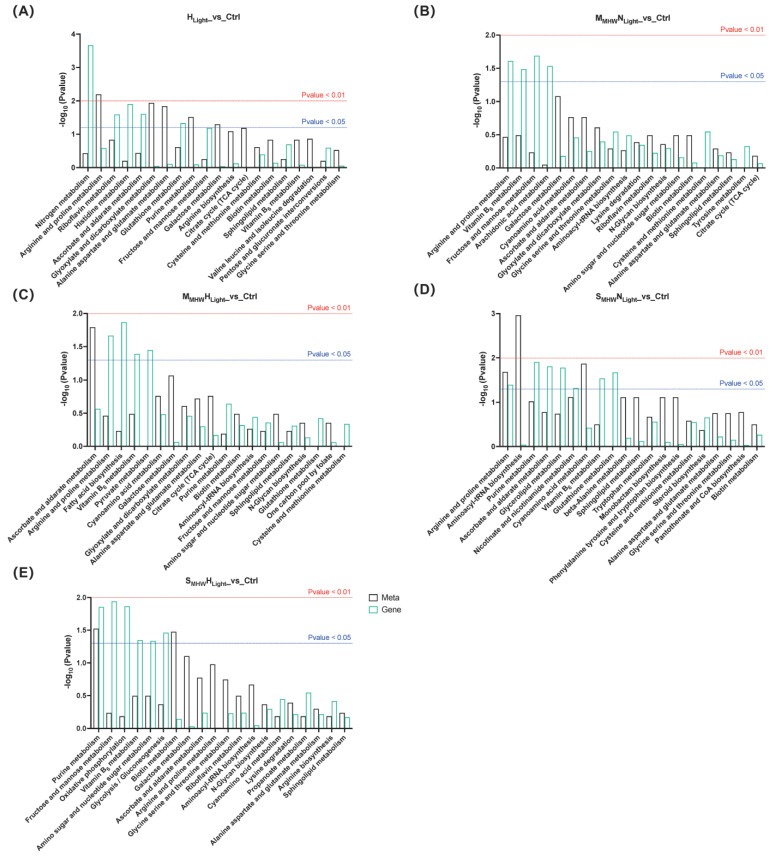
Integrated KEGG enrichment analysis of differentially expressed genes and differentially expressed metabolites in *Undaria pinnatifida* under different marine heatwave and light treatments. (**A**–**E**) correspond to H_Light__vs_Ctrl, M_MHW_N_Light__vs_Ctrl, M_MHW_H_Light__vs_Ctrl, S_MHW_N_Light__vs_Ctrl, and S_MHW_H_Light__vs_Ctrl, respectively. Black bars represent the metabolome; green bars represent the transcriptome. The red and blue dashed lines indicate significance thresholds of *p* < 0.01 and *p* < 0.05, respectively.

Integrated KEGG pathway enrichment analysis was conducted on DEGs and DEMs from the five comparison groups during the recovery phase, and the top 20 significantly enriched results for each comparison group are presented (Figure 6). Across all comparison groups, ascorbate and aldarate metabolism, arginine and proline metabolism, vitamin B_6_ metabolism, biotin metabolism, alanine, aspartate and glutamate metabolism, and sphingolipid metabolism pathways were commonly enriched in both the transcriptome and metabolome.

## 3. Discussion

### 3.1. Adaptive Responses of U. pinnatifida Under Moderate MHW Conditions

Upon exposure to moderate MHW conditions, *U. pinnatifida* exhibited a significant increase in RGR during the peak phase, accompanied by coordinated enhancements in F_v_/F_m_ and Y(II). These changes suggest that the photosynthetic system rapidly adjusted to short-term thermal stress. This response pattern is similar to that observed in *Sargassum fusiforme* under elevated temperatures [37]. The increased levels of photosynthetic pigments (Chl *a*, Chl *c*, and Fux) during the peak phase coincided with the maintenance of high photosynthetic performance, suggesting an optimization of pigment composition to potentially enhance light harvesting capacity under these conditions, contributing to the overall adaptive response observed [38]. During the cooling phase, F_v_/F_m_ and Y(II) declined slightly, reflecting a transient inhibitory effect induced by the MHW. However, a significant increase in qP during the recovery phase, along with the simultaneous rebound of other chlorophyll fluorescence parameters, indicated that *U. pinnatifida* was able to restore photosynthetic function by improving photochemical efficiency. Meanwhile, the marked elevation in Y(NPQ) during recovery and the significant enrichment of the carotenoid biosynthesis pathway suggest that the organism enhanced non-photochemical quenching via the regulation of the carotenoid cycle, thereby effectively dissipating the excess excitation energy [39]. Notably, the rETR of the moderate MHW exposure remained consistently higher than that of the control group, indicating the sustained activity of the electron transport chain. In addition, the accumulation of citric acid and α-ketoglutaric acid likely provided essential carbon skeletons and energy intermediates to support the enhanced photosynthetic processes, reflecting the improved carbon assimilation efficiency [40,41]. These regulatory mechanisms at the photosynthetic level may have acted in concert with antioxidant defenses to maintain physiological homeostasis under MHW conditions.

Despite the growth-promoting trend observed under moderate MHW stress, *U. pinnatifida* established a well-coordinated antioxidant defense system. Throughout the exposure period, TAC and the activities of antioxidant enzymes such as SOD, CAT, and POD remained consistently higher than those in the control group. At the transcript level, the *SOD1* gene was significantly upregulated and biological processes related to oxidoreductase activity were significantly enriched, suggesting that enzymatic antioxidant pathways were actively engaged in scavenging reactive oxygen species (ROS). Similar preadaptive defense mechanisms have also been reported in other macroalgal species [42,43]. Metabolomic data further supported the existence of this defense system. Metabolites such as ascorbic acid, pyridoxine, 4-pyridoxate, and pyridoxamine were upregulated, and integrated analysis revealed the upregulation of the vitamin B_6_ metabolism pathway, suggesting that *U. pinnatifida* also activated non-enzymatic antioxidant mechanisms [44]. Recent studies have shown that vitamin B_6_, in addition to its conventional role as a coenzyme, also possesses notable non-enzymatic antioxidant activity, especially in quenching singlet oxygen. In this regard, its efficiency has been reported to exceed that of conventional antioxidants like vitamins C and E [45,46]. This multi-level antioxidant response effectively limited oxidative damage, as evidenced by the transient increase in the MDA content during the cooling phase and its rapid decline during the recovery phase. The parallel recovery patterns in Y(II) and RGR as MDA levels normalized further demonstrate the effectiveness of antioxidant protection. MDA serves as a reliable indicator of oxidative stress-induced membrane damage that directly impacts photosynthetic membranes and associated complexes [47], making the limited MDA accumulation particularly significant for maintaining physiological performance under MHW stress. The rise in the proline levels during the cooling phase, together with the significant enrichment of the arginine and proline metabolism pathway, further suggests a coordinated response between osmotic regulation and antioxidant defense [48,49].

### 3.2. Stress Responses and Partial Recovery of U. pinnatifida Under Severe MHW Conditions

Under severe MHW stress, *U. pinnatifida* exhibited a response pattern distinct from that under moderate MHWs, with notable differences in physiological responses, damage extent, and recovery capacity. The temporary increase in RGR during the peak phase, accompanied by enhanced Y(II) and rETR_max_ values, indicated a compensatory stress response. This phenomenon likely occurred because *U. pinnatifida*, as an opportunistic invasive species, efficiently exploited short-term favorable conditions before reaching its physiological temperature threshold [28,50]. However, such an apparently positive response could mask the accumulation of underlying cellular damage. In the cooling phase, the photosystem function declined markedly. Decreases in Y(II) and qP suggested delayed damage to the photosynthetic reaction centers [51]. Meanwhile, the increase in Y(NO) implied a disruption of photoprotective mechanisms, as cells were unable to dissipate excess energy through conventional thermal dissipation pathways, potentially exacerbating photodamage [52]. Although photosynthetic parameters such as Y(II) and qP improved during the recovery phase compared to the cooling phase, the persistently high Y(NO) further indicated that the photosystem did not recover to control levels. The sustained low levels of rETR and rETR_max_ also confirmed the long-term impairment of the electron transport chain [53]. The failure of the photoprotective mechanisms may have stemmed from energy exhaustion caused by overcompensatory responses during the peak phase. The significant downregulation of energy-related metabolites, such as NMN, adenosine, and 3′-AMP, confirmed the occurrence of disrupted energy metabolism [54,55]. Photosynthetic pigment components exhibited divergent recovery dynamics: the Chl *c* and Fux contents decreased significantly during the cooling phase and partially recovered during the recovery phase but did not return to the control levels, while the Chl *a* content only began to decline in the recovery phase. These results indicate that the initial impairment of photosynthetic efficiency induced by severe MHW is primarily attributable to direct damage to the photosystem machinery and the electron transport chain, or to impaired repair mechanisms, rather than being solely caused by pigment loss. Furthermore, although the pigment system retained some regulatory capacity, it was insufficient to restore homeostasis within a short period. Similar findings were reported in *Zonaria turneriana*, where pigment levels declined consistently under extreme MHWs (28 °C) [56].

The cellular damage induced by severe MHWs was accompanied by a series of complex physiological responses. The significant downregulation of multiple lysophosphatidylcholine metabolites, along with the specific enrichment of 20:4-LPA and 20:5-LPA, indicated sustained injury to the cellular membrane system. Such widespread alterations in membrane lipid composition not only reduced membrane fluidity and stability but may also have triggered subsequent oxidative damage [57]. The significant enrichment of the glycerolipid metabolism pathway suggested that cells attempted to maintain membrane lipid homeostasis through transcriptional regulation, although this compensatory mechanism appeared insufficient to repair the damage within the experimental timeframe [58]. A vicious cycle likely developed between membrane damage and oxidative stress. Lipid peroxidation exacerbated ROS production, while the elevated ROS levels further compromised membrane integrity. The proline and MDA levels increased significantly during the cooling phase, and although they declined during recovery, both remained above control levels, indicating that oxidative damage was not fully resolved [59]. The function of the antioxidant system displayed a clear temporal variation. The TAC decreased significantly during the cooling phase but recovered notably during the recovery phase, suggesting the gradual restoration of total antioxidant capacity. However, the SOD and CAT activities remained suppressed in both phases, and the POD activity declined significantly only during recovery. Although antioxidant-related molecular functions were enriched, the transcriptional response did not translate into full enzymatic recovery within a short period. This implies that the specific components of the antioxidant defense system may require longer to recover, or that cells had shifted toward reliance on non-enzymatic antioxidant pathways to alleviate oxidative stress. Similar findings have been reported in *Ulva prolifera* (Chlorophyta), where the SOD activity decreased significantly and the MDA content increased under heat stress [60]. Significant changes in the amino acid levels, such as L-tryptophan and L-arginine, indicated that oxidative damage had impacted the amino acid metabolism, reflecting stress-induced metabolic adjustments [61]. Enrichment of the valine, leucine, and isoleucine degradation pathway suggested that branched-chain amino acids were catabolized to support energy metabolism and mitigate oxidative stress [62,63,64]. The upregulation of γ-Glu-Cys, a precursor of glutathione, suggested an attempt to enhance non-enzymatic antioxidant capacity [65]. Additionally, the abnormal accumulation of indole and 5-HIAA indicated that metabolic disruptions had extended to the level of secondary metabolism.

Unlike the linear physiological responses typically induced by conventional temperature stress, the damage caused by severe MHWs exhibited pronounced delay and persistence, reflecting a distinct adaptive strategy of *U. pinnatifida* in response to short-term extreme temperature fluctuations. Although a certain degree of self-repair was observed, parameters did not return to control levels, at least within the experimental observation period. Understanding this response pattern is crucial for predicting the long-term impacts of MHW events on the population dynamics and ecological functions of *U. pinnatifida*, as negative effects may persist well beyond the return of favorable environmental conditions.

### 3.3. Combined Stress Effects of MHWs and High Light on U. pinnatifida

High light intensities exacerbated the inhibitory effects of MHWs on the growth and photosynthetic performance of *U. pinnatifida*. Multiple studies have reported that light intensity influences the sensitivity of plants to temperature stress [66,67,68]. Compared with the MHW treatment alone, the RGR in the combined high light and MHW groups showed a consistent downward trend under both moderate and severe MHW conditions. This growth inhibition was closely associated with disturbances in the photosynthetic function. The decline in Y(II), accompanied by a marked increase in Y(NPQ), indicated that an excessive light input forced cells to dissipate more energy via heat dissipation pathways [69,70]. Under the moderate MHW with high light treatment, such protective regulation remained functional. However, in the severe MHW with the high light condition, a sharp increase in Y(NO) during the cooling phase revealed the severe disruption of the photosynthetic electron transport chain [71]. The dysfunction of the photosynthetic system was directly reflected at the level of energy metabolism. The significant enrichment of the oxidative phosphorylation and citrate cycle pathways, along with the downregulation of energy-related metabolites such as adenosine and GMP, suggested that *U. pinnatifida* attempted to compensate for energy shortages by adjusting energy metabolism. However, this compensation was inefficient under combined high light and MHW stress [72,73]. Under the severe MHW with high light treatment, the downregulation of *Lhca1* and upregulation of *Lhca4* indicated a reorganization of the light-harvesting complex, possibly as an acclimatory response to the excessive light conditions. The continuous decline in pigment content, together with the significant downregulation of vitamin B_2_, further confirmed the serious damage to the light-harvesting system. Vitamin B_2_ has been shown to play an important role as a photosynthetic cofactor in mitigating abiotic stress [74,75,76]. The general decline in α, along with the pronounced decrease in rETR, revealed an overall reduction in light utilization efficiency [77].

Compared to MHW treatment alone, the antioxidant defense system of *U. pinnatifida* was more severely suppressed under the combined high light conditions. The TAC was significantly reduced, especially under the severe MHW with high light, and the antioxidant enzyme activities continued to decline. This weakened antioxidant capacity directly resulted in persistently elevated MDA levels, indicating sustained lipid peroxidation and membrane damage. The increased proline content and significant upregulation of metabolites such as D-sorbitol suggested that *U. pinnatifida* attempted to accumulate osmoprotectants in response to stress, but failed to effectively maintain cellular homeostasis [78,79]. Related studies have shown that in tropical seagrasses such as *Enhalus acoroides* (Angiosperm), exposure to high temperature and hypoxia can lead to electron accumulation, excessive ROS production, imbalance of antioxidant systems, and irreversible damage to the photosynthetic apparatus under oxidative stress [80]. These results indicate that the combined effect of high light and MHWs is not merely additive, but rather a synergistic reinforcement through specific molecular mechanisms, leading to more severe physiological suppression and metabolic disruption than either stressor alone. Such compound stress responses have important implications for predicting the population dynamics of *U. pinnatifida* under future climate change scenarios, especially considering that MHW events in natural environments often co-occur with changes in light availability.

## 4. Materials and Methods

### 4.1. Sample Collection and Processing

Young sporophytes of *U. pinnatifida* (50 ± 5 cm in length) were collected in December 2023 from a seaweed cultivation site in Lidao, Rongcheng, Shandong Province, China (36.822° N, 122.327° E). The thalli were transported to the laboratory under dark and cooled conditions to minimize physiological stress. Upon arrival, thalli were rinsed with sterile, filtered seawater to remove epiphytes and debris. Approximately 2000 discs (1 cm diameter) were excised from meristematic regions using a sterile cork borer and acclimated for three days under controlled conditions (10 °C, 90 μmol photons m^−2^ s^−1^, 12:12 h light/dark, continuous aeration) in sterile seawater supplemented with one-quarter-strength PESI. The medium was renewed daily.

### 4.2. Experimental Design

Temperature anomalies of +3 °C and +6 °C relative to the baseline were used to represent moderate and severe MHW conditions, respectively, based on the MHW classification framework [2] and regional characteristics of the Northwest Pacific [81,82]. The experimental baseline temperature was set at 10 °C. In the moderate MHW treatment, the temperature was gradually increased by 0.5 °C per day until reaching 13 °C (warming phase), whereas the severe MHW treatment applied a ramping rate of 1 °C per day to reach 16 °C (warming phase). Once target temperatures were attained, conditions were maintained isothermally for 7 days (peak phase), followed by a temperature decrease at the same rates until returning to 10 °C (cooling phase). Thereafter, all treatments were maintained at 10 °C for an additional 7 days (recovery phase).

The experiment followed a fully factorial design, incorporating three MHW intensities (control: 10 °C; moderate MHW: +3 °C; severe MHW: +6 °C) and two light intensities (normal: 90 μmol photons m^−2^ s^−1^; high: 270 μmol photons m^−2^ s^−1^), resulting in six treatment combinations. Each treatment was conducted in triplicate.

### 4.3. Culture Conditions

Following acclimation, healthy discs were randomly distributed across treatments at a fresh weight of 1 g L^−1^. Cultures were maintained under a 12:12 h light/dark cycle in sterile seawater enriched with one-quarter-strength PESI, with medium renewal being performed every 72 h. Continuous aeration was provided, and environmental parameters (temperature and light) were recorded hourly using calibrated sensors. Sampling for physiological and biochemical measurements occurred on days 6, 13, 19, and 26, corresponding to the warming, peak, cooling, and recovery phases, respectively. Samples collected on day 26 were also used for transcriptomic and metabolomic analyses.

### 4.4. Measurement of Growth

Fresh weight was recorded at the beginning of the experiment and at each sampling point after blotting the discs to remove surface moisture. The relative growth rate (RGR; % day^−1^) was calculated as follows:

RGR = 100 × (ln W_t_ − ln W_0_)/t (1)

where W_0_ is the initial fresh weight, W_t_ is the fresh weight at sampling, and t is the duration in days.

### 4.5. Measurement of Pigment Content

Approximately 0.25 g of algal tissue was blotted dry and weighed. Pigments were extracted sequentially in 2 mL dimethyl sulfoxide (DMSO) and 3 mL acetone under dark conditions. After a 5 min DMSO incubation, the extract was combined with 0.5 mL distilled water and measured at 665, 631, 582, and 480 nm. Residual tissue was further extracted in acetone for 2 h, after which methanol and water were added, and measured at 664, 631, 581, and 470 nm. The concentrations of chlorophyll *a* (Chl *a*), chlorophyll *c* (Chl *c*), and fucoxanthin (Fux) (mg g^−1^ FW) were calculated based on absorbance measurements and equations described in the literature [83].

### 4.6. Measurement of Chlorophyll Fluorescence Parameters

Chlorophyll fluorescence parameters were measured using a Dual-PAM-100 system (Walz, Effeltrich, Germany). Samples were dark-adapted for 20 min prior to measurement. Fluorescence signals were recorded under standard actinic light using the Fluo + P700 mode and the SP Analysis module. Key parameters derived from the Slow Kinetics module included the maximum quantum yield of PSII photochemistry (F_v_/F_m_), the photochemical quenching coefficient (qP), the effective quantum yield of PSII [Y(II)], the quantum yield of regulated energy dissipation [Y(NPQ)], and the quantum yield of non-regulated energy dissipation [Y(NO)]. Rapid light–response curves were generated using the Light Curve program, with stepwise increases in irradiance. The relationship between the relative electron transport rate (rETR) and photosynthetically active radiation (PAR) was determined using least squares regression to obtain the maximum electron transport rate (rETR_max_), initial slope (α), and saturating irradiance (I_k_).

### 4.7. Measurement of Biochemical Indicators

After each sampling point, algal tissues were snap-frozen in liquid nitrogen and homogenized in pre-chilled 0.1 M PBS (pH 7.4, 1:9 *w*/*v*) at 70 Hz for 1 min. Homogenates were centrifuged at 6000 rpm for 10 min at 4 °C, and the supernatants were collected. The total antioxidant capacity (TAC), proline content, malondialdehyde (MDA) content, and activities of superoxide dismutase (SOD), catalase (CAT), and peroxidase (POD) were determined using kits (Nanjing Jiancheng, Nanjing, China) according to the manufacturer’s protocols.

### 4.8. De Novo Transcriptomic Profiling and Analysis

At the end of the experiment, approximately 100 mg of algal tissue was collected per treatment. RNA was extracted using the Plant RNA Extraction Kit (Qiagen, Hilden, Germany) with DNase I treatment (Takara, Osaka, Japan), and its quality (RIN ≥ 8.0), purity (A260/A280 = 1.8–2.0; A260/A230 > 2.0), and concentration were subsequently assessed. Strand-specific libraries were prepared using the NEBNext Ultra II RNA Library Prep Kit (NEB, Ipswich, MA, USA) and sequenced on an Illumina NovaSeq 6000 platform (Illumina, San Diego, CA, USA) (PE150), generating at least 6 Gb per sample. Clean reads were filtered using fastp v0.23.1, quality-checked with FastQC v0.11.9, and assembled de novo using Trinity v2.13.2. Redundant transcripts were removed using CD-HIT v4.8.1 (95% identity), and coding regions were predicted with TransDecoder v5.5.0. Annotation was performed using DIAMOND v2.0.15, hmmscan (HMMER v3.3.2), eggNOG-mapper v2.1.9, KAAS, and Blast2GO v5.2, by referencing Nr, Swiss-Prot, Pfam, KOG, eggNOG, Kyoto Encyclopedia of Genes and Genomes (KEGG), and Gene Ontology (GO) databases. Transcript abundance was quantified with Salmon v1.9.0 and summarized at the gene level with tximport v1.22.0. Differential expression analysis was performed using DESeq2 v1.36.0 (|log_2_ fold change (FC)| ≥ 1, adjusted *p* < 0.05). GO and KEGG enrichment analyses were conducted using clusterProfiler v4.6.0.

### 4.9. Quantitative Real-Time PCR

Total RNA was extracted from recovery-phase samples using a plant RNA kit (Tiangen, Beijing, China), and reverse transcription was performed with a gDNA removal kit (Takara, Osaka, Japan). Primers were designed with Primer Premier v6.0. qRT-PCR was performed using TB Green chemistry on a CFX96 system (Bio-Rad, Hercules, CA, USA) under standard cycling conditions. Relative expression levels were calculated using the 2^−ΔΔCT^ method, normalized to *eEF1β*. Table S1 summarizes all primer sequences used in this study. Six biological and three technical replicates were used per treatment.

### 4.10. Untargeted Metabolomic Profiling and Analysis

Untargeted metabolomic profiling was conducted using liquid chromatography–mass spectrometry (LC-MS). At the end of the experiment, approximately 200 mg of algal tissue was sampled per treatment, frozen in liquid nitrogen, and stored at −80 °C. Metabolites were extracted and six biological replicates plus the pooled QC samples were included. LC-MS was performed using a Vanquish Flex UHPLC and Q Exactive HF-X MS system (Thermo Scientific, Waltham, MA, USA). Data were processed in Compound Discoverer v3.2. The matrix was normalized to internal standards, Pareto-scaled, and analyzed in MetaboAnalyst 5.0 via principal component analysis (PCA) and partial least squares discriminant analysis (PLS-DA). DEMs were screened based on VIP > 1.5, *p* < 0.05, and FC ≥ 2 or ≤0.5. Clustering and pathway enrichment (KEGG, *p* < 0.05) were also performed.

### 4.11. Integrated KEGG Enrichment Analysis of Transcriptomic and Metabolomic Profiles

A KEGG-based joint pathway analysis was conducted to explore the functional overlap between the transcriptomic and metabolomic datasets. Gene and metabolite profiles were independently filtered and scaled. Transcript levels (FPKM) were log_2_-transformed and standardized; metabolite intensities were adjusted for batch effects and normalized. Both datasets were annotated using KEGG, and shared pathways were identified (*p* < 0.05).

### 4.12. Statistical Analysis and Data Visualization

Three-way repeated-measures ANOVA was used to assess the effects of MHW intensity, light intensity, and sampling time on growth and physiological traits. Data from physiological, transcriptomic, and metabolomic analyses were analyzed and visualized in R v3.6.3, Python v3.12, and GraphPad Prism v10.2.0. Figures were finalized and formatted in Adobe Illustrator v24.2.3.

## 5. Conclusions

In summary, *U. pinnatifida* exhibited adaptive responses under moderate MHW conditions, as reflected by enhanced photosynthetic performance, improved pigment accumulation, and sustained carbon and energy metabolism, all of which contributed to the maintenance of physiological homeostasis. These responses were supported by a preadaptive antioxidant defense system that effectively mitigated oxidative stress. In contrast, severe MHWs caused widespread physiological disruption, with an initial compensatory phase rapidly giving way to systemic dysfunction, including impaired photoprotection, metabolic imbalance, and structural damage. These effects persisted beyond the stress period, indicating delayed damage and potential long-term consequences. Moreover, co-occurring high light intensity further amplified the negative impacts, aggravating energy conversion failure and oxidative damage. Elucidating its response mechanisms under MHW stress is essential for understanding its adaptive strategies to extreme environments and provides a theoretical basis for improving cultivation management under future climate change scenarios.

## Data Availability

The original contributions presented in this study are included in the article/Supplementary Materials. Further inquiries can be directed to the corresponding authors.

## Supplementary Materials

The following supporting information can be downloaded at: https://www.mdpi.com/article/10.3390/plants14101419/s1, Figure S1: Changes in differentially expressed genes of *Undaria pinnatifida*; Figure S2: Go enrichment analysis of differentially expressed genes in *Undaria pinnatifida*; Figure S3: Gene expression of *Undaria pinnatifida* under different marine heatwave and light treatments; Figure S4: Quality control of *Undaria pinnatifida* metabolome data under marine heatwave and light treatments (positive and negative ions combined); Table S1: qRT-PCR primer sequences; Table S2: Three-way repeated measures ANOVA on physiological and biochemical indicators of *Undaria pinnatifida*; Table S3: Simple effects of marine heatwave intensity on physiological and biochemical indicators of *Undaria pinnatifida*, with light intensity and sampling time held constant; Table S4: Simple effects of light intensity on physiological and biochemical indicators of *Undaria pinnatifida*, with marine heatwave intensity and sampling time held constant; Table S5: Simple effects of sampling time on physiological and biochemical indicators of *Undaria pinnatifida*, with marine heatwave intensity and light intensity held constant; Table S6: Simple effects of marine heatwave intensity at each light intensity on physiological and biochemical indicators of *Undaria pinnatifida*; Table S7: Data quality of *Undaria pinnatifida* transcriptome under different marine heatwave and light treatments; Table S8: Single-gene functional annotation of *Undaria pinnatifida* transcriptome in seven databases under different marine heatwave and light treatments.

## References

[1] Frölicher T.L., Fischer E.M., Gruber N. (2018). Marine Heatwaves under Global Warming. Nature.

[2] Hobday A., Oliver E., Sen Gupta A., Benthuysen J., Burrows M., Donat M., Holbrook N., Moore P., Thomsen M., Wernberg T. (2018). Categorizing and Naming Marine Heatwaves. Oceanography.

[3] Holbrook N.J., Scannell H.A., Sen Gupta A., Benthuysen J.A., Feng M., Oliver E.C.J., Alexander L.V., Burrows M.T., Donat M.G., Hobday A.J. (2019). A Global Assessment of Marine Heatwaves and Their Drivers. Nat. Commun..

[4] Oliver E.C.J., Burrows M.T., Donat M.G., Sen Gupta A., Alexander L.V., Perkins-Kirkpatrick S.E., Benthuysen J.A., Hobday A.J., Holbrook N.J., Moore P.J. (2019). Projected Marine Heatwaves in the 21st Century and the Potential for Ecological Impact. Front. Mar. Sci..

[5] Smale D.A. (2019). Impacts of Ocean Warming on Kelp Forest Ecosystems. New Phytol..

[6] Thomsen M.S., Mondardini L., Alestra T., Gerrity S., Tait L., South P.M., Lilley S.A., Schiel D.R. (2019). Local Extinction of Bull Kelp (*Durvillaea* spp.) Due to a Marine Heatwave. Front. Mar. Sci..

[7] Smale D.A., Pessarrodona A., King N., Burrows M.T., Yunnie A., Vance T., Moore P. (2020). Environmental Factors Influencing Primary Productivity of the Forest-Forming Kelp *Laminaria hyperborea* in the Northeast Atlantic. Sci. Rep..

[8] Coleman M.A., Wernberg T. (2020). The Silver Lining of Extreme Events. Trends Ecol. Evol..

[9] Filbee-Dexter K., Wernberg T. (2018). Rise of Turfs: A New Battlefront for Globally Declining Kelp Forests. Bioscience.

[10] Arafeh-Dalmau N., Schoeman D.S., Montaño-Moctezuma G., Micheli F., Rogers-Bennett L., Olguin-Jacobson C., Possingham H.P. (2020). Marine Heat Waves Threaten Kelp Forests. Science.

[11] Wernberg T., Smale D.A., Tuya F., Thomsen M.S., Langlois T.J., De Bettignies T., Bennett S., Rousseaux C.S. (2013). An Extreme Climatic Event Alters Marine Ecosystem Structure in a Global Biodiversity Hotspot. Nat. Clim. Change.

[12] Wernberg T., Bennett S., Babcock R.C., de Bettignies T., Cure K., Depczynski M., Dufois F., Fromont J., Fulton C.J., Hovey R.K. (2016). Climate-Driven Regime Shift of a Temperate Marine Ecosystem. Science.

[13] Vergés A., McCosker E., Mayer-Pinto M., Coleman M.A., Wernberg T., Ainsworth T., Steinberg P.D. (2019). Tropicalisation of Temperate Reefs: Implications for Ecosystem Functions and Management Actions. Funct. Ecol..

[14] Pessarrodona A., Filbee-Dexter K., Alcoverro T., Boada J., Feehan C.J., Fredriksen S., Grace S.P., Nakamura Y., Narvaez C.A., Norderhaug K.M. (2021). Homogenization and Miniaturization of Habitat Structure in Temperate Marine Forests. Glob. Chang. Biol..

[15] Duarte C.M., Gattuso J., Hancke K., Gundersen H., Filbee-Dexter K., Pedersen M.F., Middelburg J.J., Burrows M.T., Krumhansl K.A., Wernberg T. (2022). Global Estimates of the Extent and Production of Macroalgal Forests. Glob. Ecol. Biogeogr..

[16] Gurgel C.F.D., Camacho O., Minne A.J.P., Wernberg T., Coleman M.A. (2020). Marine Heatwave Drives Cryptic Loss of Genetic Diversity in Underwater Forests. Curr. Biol..

[17] Smyth T. (2011). Penetration of UV Irradiance into the Global Ocean. J. Geophys. Res. Ocean..

[18] Helmuth B., Broitman B.R., Yamane L., Gilman S.E., Mach K., Mislan K., Denny M.W. (2010). Organismal Climatology: Analyzing Environmental Variability at Scales Relevant to Physiological Stress. J. Exp. Biol..

[19] Harley C.D., Anderson K.M., Demes K.W., Jorve J.P., Kordas R.L., Coyle T.A., Graham M.H. (2012). Effects of Climate Change on Global Seaweed Communities. J. Phycol..

[20] Xiao X., De Bettignies T., Olsen Y.S., Agusti S., Duarte C.M., Wernberg T. (2015). Sensitivity and Acclimation of Three Canopy-Forming Seaweeds to UVB Radiation and Warming. PLoS ONE.

[21] Nepper-Davidsen J., Andersen D., Pedersen M. (2019). Exposure to Simulated Heatwave Scenarios Causes Long-Term Reductions in Performance in *Saccharina latissima*. Mar. Ecol. Prog. Ser..

[22] Heinrich S., Valentin K., Frickenhaus S., John U., Wiencke C. (2012). Transcriptomic Analysis of Acclimation to Temperature and Light Stress in *Saccharina Latissima* (Phaeophyceae). PLoS ONE.

[23] Zhang Y., Xiao Z., Wei Z., Long L. (2024). Increased Light Intensity Enhances Photosynthesis and Biochemical Components of Red Macroalga of Commercial Importance, *Kappaphycus Alvarezii*, in Response to Ocean Acidification. Plant Physiol. Biochem..

[24] Delebecq G., Davoult D., Menu D., Janquin M.-A., Dauvin J.-C., Gevaert F. (2013). Influence of Local Environmental Conditions on the Seasonal Acclimation Process and the Daily Integrated Production Rates of *Laminaria Digitata* (Phaeophyta) in the English Channel. Mar. Biol..

[25] Bischof K., Gomez I., Molis M., Hanelt D., Karsten U., Lüder U., Roleda M.Y., Zacher K., Wiencke C. (2006). Ultraviolet Radiation Shapes Seaweed Communities. Rev. Environ. Sci. Bio./Technol..

[26] Heinrich S., Valentin K., Frickenhaus S., Wiencke C. (2015). Temperature and Light Interactively Modulate Gene Expression in *Saccharina latissima* (Phaeophyceae). J. Phycol..

[27] Gao G., Liu Y., Li X., Feng Z., Xu J. (2016). An Ocean Acidification Acclimatised Green Tide Alga Is Robust to Changes of Seawater Carbon Chemistry but Vulnerable to Light Stress. PLoS ONE.

[28] Epstein G., Smale D.A. (2017). *Undaria pinnatifida*: A Case Study to Highlight Challenges in Marine Invasion Ecology and Management. Ecol. Evol..

[29] South P.M., Floerl O., Forrest B.M., Thomsen M.S. (2017). A Review of Three Decades of Research on the Invasive Kelp *Undaria pinnatifida* in Australasia: An Assessment of Its Success, Impacts and Status as One of the World’s Worst Invaders. Mar. Environ. Res..

[30] Buschmann A.H., Camus C., Infante J., Neori A., Israel Á., Hernández-González M.C., Pereda S.V., Gomez-Pinchetti J.L., Golberg A., Tadmor-Shalev N. (2017). Seaweed Production: Overview of the Global State of Exploitation, Farming and Emerging Research Activity. Eur. J. Phycol..

[31] Sfriso A., Facca C. (2013). Annual Growth and Environmental Relationships of the Invasive Species *Sargassum muticum* and *Undaria pinnatifida* in the Lagoon of Venice. Estuar. Coast. Shelf Sci..

[32] Marzocchi M., Badocco D., Piovan A., Pastore P., Di Marco V., Filippini R., Caniato R. (2016). Metals in *Undaria pinnatifida* (Harvey) Suringar and *Sargassum muticum* (Yendo) Fensholt Edible Seaweeds Growing around Venice (Italy). J. Appl. Phycol..

[33] Morelissen B., Dudley B., Phillips N. (2016). Recruitment of the Invasive Kelp *Undaria pinnatifida* Does Not Always Benefit from Disturbance to Native Algal Communities in Low-Intertidal Habitats. Mar. Biol..

[34] Kim J.K., Yarish C., Hwang E.K., Park M., Kim Y. (2017). Seaweed Aquaculture: Cultivation Technologies, Challenges and Its Ecosystem Services. Algae.

[35] Watanabe Y., Nishihara G.N., Tokunaga S., Terada R. (2014). The Effect of Irradiance and Temperature Responses and the Phenology of a Native Alga, *Undaria pinnatifida* (Laminariales), at the Southern Limit of Its Natural Distribution in Japan. J. Appl. Phycol..

[36] Montie S., Thomsen M.S. (2023). Long-term Community Shifts Driven by Local Extinction of an Iconic Foundation Species Following an Extreme Marine Heatwave. Ecol. Evol..

[37] Zuo X., Xu L., Luo L., Zeng Y., Ma Z., Wu M., Chen B. (2023). Physiological Responses of *Sargassum fusiforme* Seedlings to High-Temperature Stress. Reg. Stud. Mar. Sci..

[38] Peres L.M.C., Gouvêa L.P., Hayden J., Burle G., Bastos E., Carneiro A., Horta P.A. (2023). Effects of Ocean Warming and Pollution on *Sargassum* Forests. Mar. Environ. Res..

[39] Fabbrizzi E., Munari M., Fraschetti S., Arena C., Chiarore A., Cannavacciuolo A., Colletti A., Costanzo G., Soler-Fajardo A., Nannini M. (2023). Canopy-Forming Macroalgae Can Adapt to Marine Heatwaves. Environ. Res..

[40] Feijão E., Gameiro C., Franzitta M., Duarte B., Caçador I., Cabrita M.T., Matos A.R. (2018). Heat Wave Impacts on the Model Diatom *Phaeodactylum tricornutum*: Searching for Photochemical and Fatty Acid Biomarkers of Thermal Stress. Ecol. Indic..

[41] Zhang N., Venn B., Bailey C.E., Xia M., Mattoon E.M., Mühlhaus T., Zhang R. (2024). Moderate High Temperature Is Beneficial or Detrimental Depending on Carbon Availability in the Green Alga *Chlamydomonas reinhardtii*. J. Exp. Bot..

[42] Li X., Meng X., Yang X., Duan D. (2023). Characterization of chlorophyll Fluorescence and Antioxidant Defense Parameters of Two *Gracilariopsis lemaneiformis* Strains under Different Temperatures. Plants.

[43] Cui J., Dai Y., Lai Y., Tan Y., Liu T. (2024). Effects of Abscisic Acid on the Physiological and Biochemical Responses of *Saccharina japonica* under High-Temperature Stress. Int. J. Mol. Sci..

[44] Panahi B., Farhadian M., Hosseinzadeh Gharajeh N., Mohammadi S.A., Hejazi M.A. (2024). Meta-Analysis of Transcriptomic Profiles in *Dunaliella tertiolecta* Reveals Molecular Pathway Responses to Different Abiotic Stresses. Funct. Plant Biol..

[45] Mooney S., Leuendorf J.-E., Hendrickson C., Hellmann H. (2009). Vitamin B_6_: A Long Known Compound of Surprising Complexity. Molecules.

[46] Asensi-Fabado M.A., Munné-Bosch S. (2010). Vitamins in Plants: Occurrence, Biosynthesis and Antioxidant Function. Trends Plant Sci..

[47] Sharma P., Jha A.B., Dubey R.S., Pessarakli M. (2012). Reactive Oxygen Species, Oxidative Damage, and Antioxidative Defense Mechanism in Plants under Stressful Conditions. J. Bot..

[48] Barati B., Gan S.-Y., Lim P.-E., Beardall J., Phang S.-M. (2019). Green Algal Molecular Responses to Temperature Stress. Acta Physiol. Plant..

[49] Gu K., Liu Y., Jiang T., Cai C., Zhao H., Liu X., He P. (2022). Molecular Response of *Ulva prolifera* to Short-Term High Light Stress Revealed by a Multi-Omics Approach. Biology.

[50] James K., Kibele J., Shears N.T. (2015). Using Satellite-Derived Sea Surface Temperature to Predict the Potential Global Range and Phenology of the Invasive Kelp *Undaria pinnatifida*. Biol. Invasions.

[51] Nauer F., Oliveira M.C., Plastino E.M., Yokoya N.S., Fujii M.T. (2022). Coping with Heatwaves: How a Key Species of Seaweed Responds to Heat Stress along Its Latitudinal Gradient. Mar. Environ. Res..

[52] Zheng Y., Xia Z., Wu J., Ma H. (2021). Effects of Repeated Drought Stress on the Physiological Characteristics and Lipid Metabolism of *Bombax ceiba* L. during Subsequent Drought and Heat Stresses. BMC Plant Biol..

[53] Chen X., Tang Y., Zhang H., Zhang X., Sun X., Zang X., Xu N. (2024). Physiological, Transcriptome, and Metabolome Analyses Reveal the Tolerance to Cu Toxicity in Red Macroalgae *Gracilariopsis lemaneiformis*. Int. J. Mol. Sci..

[54] Coutinho I.D., Henning L.M.M., Döpp S.A., Nepomuceno A., Moraes L.A.C., Marcolino-Gomes J., Richter C., Schwalbe H., Colnago L.A. (2018). Flooded Soybean Metabolomic Analysis Reveals Important Primary and Secondary Metabolites Involved in the Hypoxia Stress Response and Tolerance. Environ. Exp. Bot..

[55] Fakhimi N., Grossman A.R. (2024). Photosynthetic Electron Flows and Networks of Metabolite Trafficking to Sustain Metabolism in Photosynthetic Systems. Plants.

[56] Wernberg T., Straub S. (2024). Low Light Exacerbates Effects of Marine Heatwaves on Seaweeds. Mar. Ecol. Prog. Ser..

[57] Henschel J.M., Andrade A.N.d., dos Santos J.B.L., da Silva R.R., da Mata D.A., Souza T., Batista D.S. (2024). Lipidomics in Plants under Abiotic Stress Conditions: An Overview. Agronomy.

[58] Jiang J.-Y., Zhu S., Zhang Y., Sun X., Hu X., Huang H., Ren L.-J. (2019). Integration of Lipidomic and Transcriptomic Profiles Reveals Novel Genes and Regulatory Mechanisms of *Schizochytrium* sp. in Response to Salt Stress. Bioresour. Technol..

[59] González-Morales S., Solís-Gaona S., Valdés-Caballero M.V., Juárez-Maldonado A., Loredo-Treviño A., Benavides-Mendoza A. (2021). Transcriptomics of Biostimulation of Plants under Abiotic Stress. Front. Genet..

[60] Fan M., Sun X., Liao Z., Wang J., Li Y., Xu N. (2018). Comparative Proteomic Analysis of *Ulva prolifera* Response to High Temperature Stress. Proteome Sci..

[61] Heinemann B. (2021). Amino Acid Metabolism Under Drought Stress in *Arabidopsis thaliana*. Ph.D. Thesis.

[62] Lacroux J., Atteia A., Brugière S., Couté Y., Vallon O., Steyer J.-P., van Lis R. (2022). Proteomics Unveil a Central Role for Peroxisomes in Butyrate Assimilation of the Heterotrophic Chlorophyte Alga *Polytomella* sp. Front. Microbiol..

[63] Shakya M., Silvester E., Rees G., Rajapaksha K.H., Faou P., Holland A. (2022). Changes to the Amino Acid Profile and Proteome of the Tropical Freshwater Microalga *Chlorella* sp. in Response to Copper Stress. Ecotoxicol. Environ. Saf..

[64] Ingrisano R., Tosato E., Trost P., Gurrieri L., Sparla F. (2023). Proline, Cysteine and Branched-Chain Amino Acids in Abiotic Stress Response of Land Plants and Microalgae. Plants.

[65] Rai G.K., Kumar P., Choudhary S.M., Singh H., Adab K., Kosser R., Magotra I., Kumar R.R., Singh M., Sharma R. (2023). Antioxidant Potential of Glutathione and Crosstalk with Phytohormones in Enhancing Abiotic Stress Tolerance in Crop Plants. Plants.

[66] Vivanco-Bercovich M., Belando-Torrentes M.D., Figueroa-Burgos M.F., Ferreira-Arrieta A., Macías-Carranza V., García-Pantoja J.A., Cabello-Pasini A., Samperio-Ramos G., Cruz-López R., Sandoval-Gil J.M. (2022). Combined Effects of Marine Heatwaves and Reduced Light on the Physiology and Growth of the Surfgrass *Phyllospadix torreyi* from Baja California, Mexico. Aquat. Bot..

[67] Bass A.V., Smith K.E., Smale D.A. (2023). Marine Heatwaves and Decreased Light Availability Interact to Erode the Ecophysiological Performance of Habitat-forming Kelp Species. J. Phycol..

[68] Jung E.M.U., Abdul Majeed N.A.B., Booth M.W., Austin R., Sinclair E.A., Fraser M.W., Martin B.C., Oppermann L.M.F., Bollen M., Kendrick G.A. (2023). Marine Heatwave and Reduced Light Scenarios Cause Species-Specific Metabolomic Changes in Seagrasses under Ocean Warming. New Phytol..

[69] Quaas T., Berteotti S., Ballottari M., Flieger K., Bassi R., Wilhelm C., Goss R. (2015). Non-Photochemical Quenching and xanthophyll Cycle Activities In Six Green Algal Species Suggest Mechanistic Differences in the Process of Excess Energy Dissipation. J. Plant Physiol..

[70] Figueroa F.L., Celis-Plá P.S.M., Martínez B., Korbee N., Trilla A., Arenas F. (2019). Yield Losses and Electron Transport Rate as Indicators of Thermal Stress in *Fucus serratus* (Ochrophyta). Algal Res..

[71] Gebara R.C., Alho L.d.O.G., Mansano A.d.S., Rocha G.S., Melão M.d.G.G. (2023). Single and Combined Effects of Zn and Al on Photosystem II of the Green Microalgae *Raphidocelis subcapitata* Assessed by Pulse-Amplitude Modulated (PAM) Fluorometry. Aquat. Toxicol..

[72] Feng L., Wang Z., Jia D., Zou X., Rao M., Huang Z., Kuang C., Ye J., Chen C., Huang C. (2023). Functional Metabolism Pathways of Significantly Regulated Genes in *Nannochloropsis Oceanica* with Various Nitrogen/Phosphorus Nutrients for CO_2_ Fixation. Sci. Total Environ..

[73] Zhou Y., Yue Y., Chen X., Wu F., Li W., Li P., Han J. (2024). Physiological-Biochemical Responses and Transcriptomic Analysis Reveal the Effects and Mechanisms of Sulfamethoxazole on the Carbon Fixation Function of *Chlorella pyrenoidosa*. Sci. Total Environ..

[74] Alboresi A., Storti M., Cendron L., Morosinotto T. (2019). Role and Regulation of Class-C Flavodiiron Proteins in Photosynthetic Organisms. Biochem. J..

[75] Huang B., Cui J., Ran Y., Chen C., Li F., Zhang Y., Li Z., Xie E. (2022). Mechanism of Macroalgae *Gracilaria bailiniae* Responding to Cadmium and Lanthanum. Front. Plant Sci..

[76] Jiadkong K., Fauzia A.N., Yamaguchi N., Ueda A. (2024). Exogenous Riboflavin (Vitamin B_2_) Application Enhances Salinity Tolerance through the Activation of Its Biosynthesis in Rice Seedlings under Salinity Stress. Plant Sci..

[77] Rocha G.S., Melão M.G.G. (2024). Does Cobalt Antagonize P Limitation Effects on Photosynthetic Parameters on the Freshwater Microalgae *Raphidocelis subcapitata* (Chlorophyceae), or Does P Limitation Acclimation Antagonize Cobalt Effects? More Questions than Answers. Environ. Pollut..

[78] Aigner S., Arc E., Schletter M., Karsten U., Holzinger A., Kranner I. (2022). Metabolite Profiling in Green Microalgae with Varying Degrees of Desiccation Tolerance. Microorganisms.

[79] Sánchez-Thomas R., Hernández-Garnica M., Granados-Rivas J.C., Saavedra E., Peñalosa-Castro I., Rodríguez-Enríquez S., Moreno-Sánchez R. (2024). Intertwining of Cellular Osmotic Stress Handling Mechanisms and Heavy Metal Accumulation. Mol. Biotechnol..

[80] Li Z., Li H., Zhang M., Zhang L., Li J., Liu J. (2025). Physiological and Molecular Responses of Tropical Seagrass *Enhalus acoroides* Exposed to Simultaneous High Temperature and Hypoxia Stress. Mar. Environ. Res..

[81] Plecha S.M., Soares P.M.M. (2020). Global Marine Heatwave Events Using the New CMIP6 Multi-Model Ensemble: From Shortcomings in Present Climate to Future Projections. Environ. Res. Lett..

[82] Xue J., Shan H., Liang J.-H., Dong C. (2023). Assessment and Projections of Marine Heatwaves in the Northwest Pacific Based on CMIP6 Models. Remote Sens..

[83] Seely G., Duncan M., Vidaver W. (1972). Preparative and Analytical Extraction of Pigments from Brown Algae with Dimethyl Sulfoxide. Mar. Biol..

