# Supplementary material for: Combined Effects of Marine Heatwaves and Light Intensity on the Physiological, Transcriptomic, and Metabolomic Profiles of Undaria pinnatifida
Source: Plants (Basel). 2025 May 9;14(10):1419. doi: 10.3390/plants14101419 (PMC12115088; doi:10.3390/plants14101419)
Supplement: Supplementary file 1 [file plants-14-01419-s001.zip › plants-3592702-supplementary.pdf]

Combined effects of marine heatwaves and light intensity on the physiological, transcriptomic, and metabolomic profiles of *Undaria pinnatifida*

Han Mo Song[1], Yan Liu[1, *], Qing Li Gong[1], Xu Gao[1, *]

[1]Key Laboratory of Mariculture (Ministry of Education), Fisheries College, Ocean University of China, Qingdao 266003, China

*Corresponding author:

Yan Liu

Email: qd_liuyan@ouc.edu.cn

Xu Gao

Email: gaoxu@ouc.edu.cn

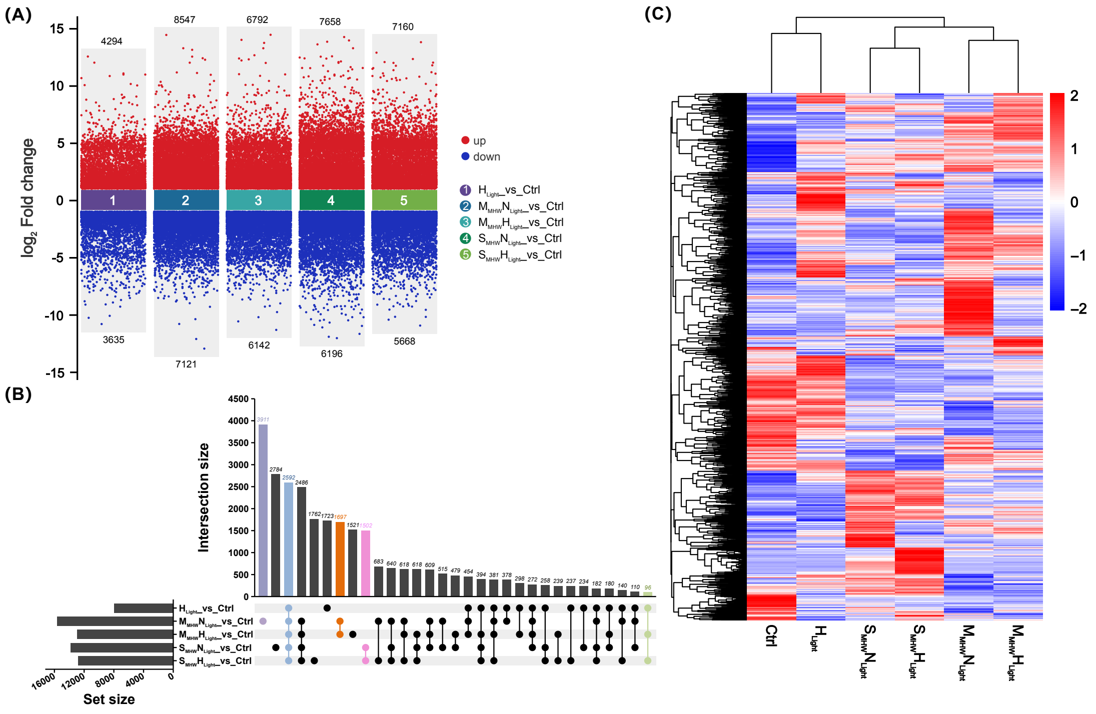

Figure S1. Changes in differentially expressed genes of *Undaria pinnatifida*. (A) DEGs distribution between treatment and control groups (red: upregulated, blue: downregulated). (B) Upset plot of DEGs, with set size indicating total DEGs per group and intersection size showing shared DEGs. (C) Heatmap of DEGs expression patterns (red: high expression, blue: low expression).

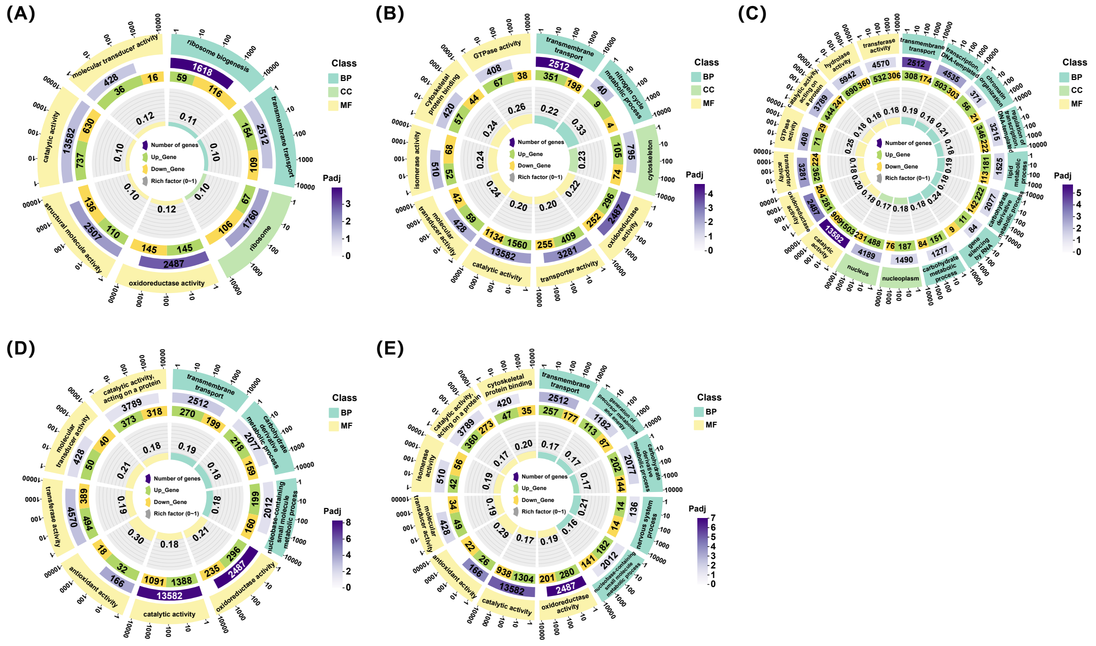

Figure S2. Go enrichment analysis of differentially expressed genes in *Undaria pinnatifida*. (A–E) GO enrichment analysis for H_{Light}_vs_Ctrl, $M_{MHW}N_{Light}$_vs_Ctrl, $M_{MHW}H_{Light}$ _vs_Ctrl, $S_{MHW}N_{Light}$_vs_Ctrl, and $S_{MHW}H_{Light}$_vs_Ctrl groups, respectively. From outer to inner circles: significantly enriched GO categories, background gene count, upregulated (green) and downregulated (yellow) gene count, and enrichment factor.

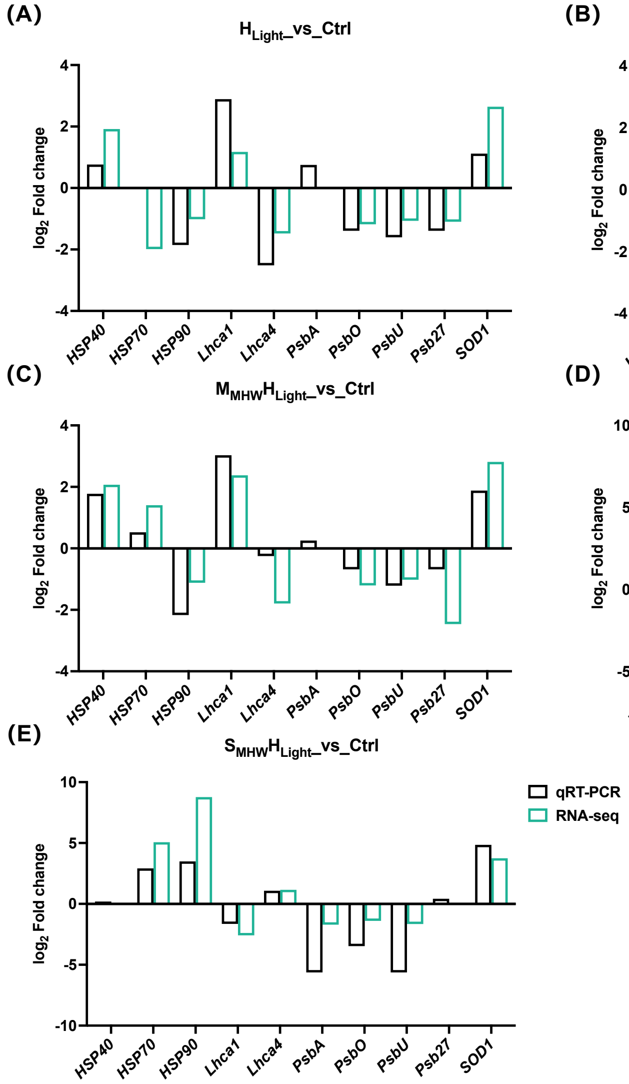

Figure S3. Gene expression of *Undaria pinnatifida* under different marine heatwave and light treatments. (A–E) qRT-PCR validation of DEGs in the H_{Light}_vs_Ctrl, $M_{MHW}N_{Light}$_vs_Ctrl, $M_{MHW}H_{Light}$_vs_ Ctrl, $S_{MHW}N_{Light}$_vs_Ctrl, and $S_{MHW}H_{Light}$_vs_Ctrl groups, respectively. Black bars represent qRT-PCR data, while green bars indicate transcriptome data.

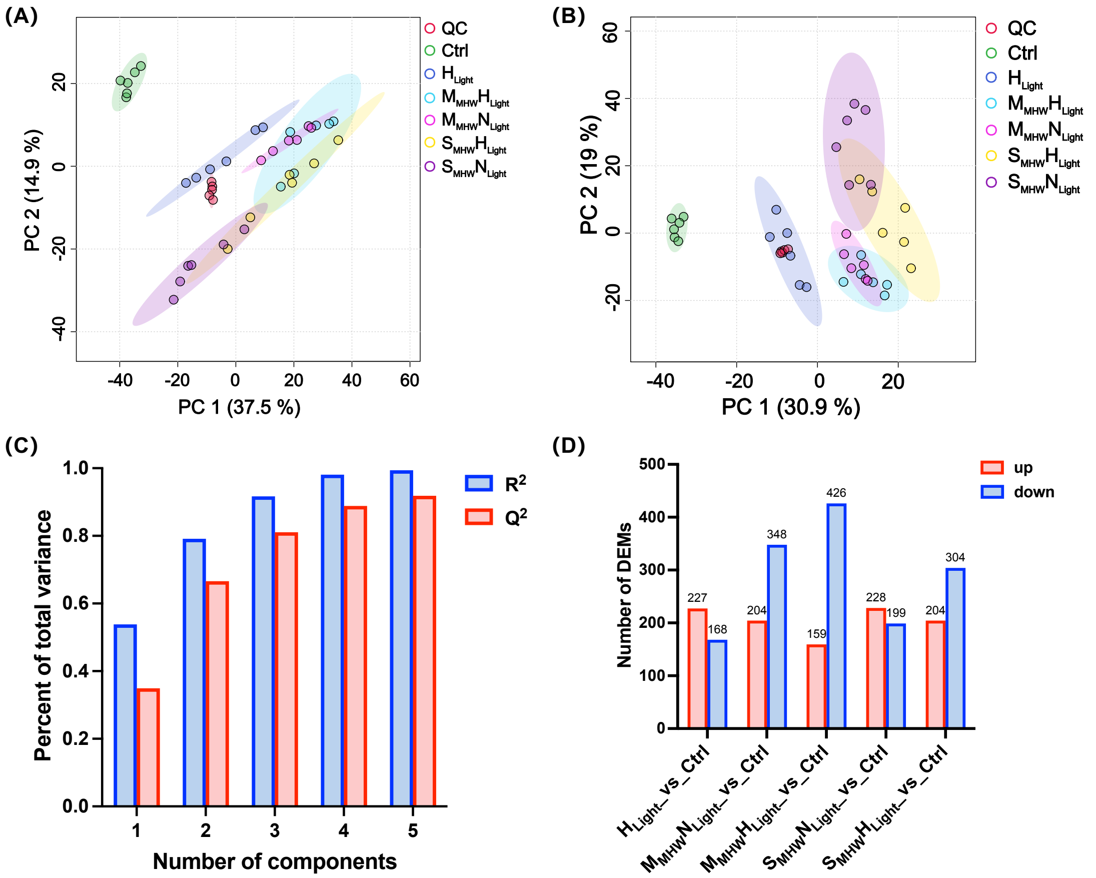

Figure S4. Quality control of *Undaria pinnatifida* metabolome data under marine heatwave and light treatments (positive and negative ions combined). (A) Principal component analysis. (B) Partial least squares discriminant analysis. (C) Statistical analysis of R^2 and Q^2. (D) Number of upregulated and downregulated differential metabolites.

Table S1. qRT-PCR primer sequences.

Gene Name	Abbreviation	Forward Primer (5'-3')	Reverse Primer (5'-3')
Heat shock protein 40	*HSP40*	GATCGCCGATGAACCTGCTGAC	AAAGCTGAAGACAGGATCACGGTTAC
Heat shock protein 70	*HSP70*	AAGCACAAGGAGATTGAGGCGATG	CGTCGTCGTCGTTCGCATCTC
Heat shock protein 90	*HSP90*	AGAAGTCGCAGTCCAGGAAGGG	TGTTGGAGTACAGCGAGTTGATGATG
Light-harvesting complex I chlorophyll a/b binding protein 1	*Lhca1*	TCACACGTTCCAAGTTCCAAGACTG	GACGAAGCCTCCGCAGCAAC
Light-harvesting complex I chlorophyll a/b binding protein 4	*Lhca4*	CTTGTACGAGGACGACTTGACGAAG	TGCTCCTTGTTTGCCGTCACG
Photosystem II protein D1	*PsbA*	GCAGCACCTCCTGTAGATATTGATGG	TTTGAACTAGGTATCACAGCACCACTG
Photosystem II oxygen-evolving enhancer protein 1	*PsbO*	TCCGCACGCCATCCTACCG	ATCTCCTTGTCTCCCTCGATTCCG
Photosystem II oxygen-evolving enhancer protein 3	*PsbU*	TCGAACGGGCCGTACTCGTC	GAATTTGCTCTCGTGCTTCTTCATCG
Photosystem II 27 kDa protein	*Psb27*	ACTCCAACCTTTACACTTCGATCAGC	GATGTCGGTGTACTCCTGCAACAG
Cu/Zn-superoxide dismutase	*SOD1*	CAGGCTCAGGAGGAAGCGCC	GTGGTCGGCATCGCTGCTTC
Reference gene	*eEF1β*	CAGTAGTCACCGTGGCTATTGC	CGGCAAACGAAACAACGGTA

Table S2. Three-way repeated measures ANOVA on physiological and biochemical indicators of *Undaria pinnatifida.* MHW, Light, and Time represent marine heatwave intensity, light intensity, and sampling time point, respectively.

Factors	MHW × Light × Time				MHW × Light				MHW × Time				Light × Time				MHW				Light				Time			
	df	F	*P*	η^2p	df	F	*P*	η^2p	df	F	*P*	η^2p	df	F	*P*	η^2p	df	F	*P*	η^2p	df	F	*P*	η^2p	df	F	*P*	η^2p
**RGR**	6	6.864	<0.001	0.534	2	14.382	0.001	0.706	6	40.559	<0.001	0.871	3	27.245	<0.001	0.694	2	83.001	<0.001	0.933	1	39.790	<0.001	0.768	3	218.618	<0.001	0.948
**F_v/F_m**	6	9.770	<0.001	0.620	2	13.602	0.001	0.694	6	12.214	<0.001	0.671	3	66.961	<0.001	0.848	2	147.064	<0.001	0.961	1	524.249	<0.001	0.978	3	176.914	<0.001	0.936
**qP**	6	1.901	0.107	0.241	2	10.100	0.003	0.627	6	37.863	0.000	0.863	3	6.880	0.001	0.364	2	34.551	<0.001	0.852	1	8.803	0.012	0.423	3	77.996	<0.001	0.867
**Y(II)**	6	3.037	0.017	0.336	2	19.617	<0.001	0.766	6	42.679	<0.001	0.877	3	5.951	0.002	0.332	2	166.069	<0.001	0.965	1	271.161	<0.001	0.958	3	230.395	<0.001	0.950
**Y(NPQ)**	6	15.250	<0.001	0.718	2	23.244	<0.001	0.795	6	18.166	<0.001	0.752	3	12.530	<0.001	0.511	2	108.772	<0.001	0.948	1	85.458	<0.001	0.877	3	60.563	<0.001	0.835
**Y(NO)**	6	15.683	<0.001	0.723	2	130.210	<0.001	0.956	6	13.997	<0.001	0.700	3	1.448	0.245	0.108	2	147.583	<0.001	0.961	1	51.896	<0.001	0.812	3	67.370	<0.001	0.849
**Y(I)**	6	9.388	<0.001	0.610	2	13.632	0.001	0.694	6	13.954	<0.001	0.699	3	8.422	<0.001	0.412	2	20.449	<0.001	0.773	1	18.168	0.001	0.602	3	130.765	<0.001	0.916
**Y(ND)**	6	3.713	0.006	0.382	2	11.844	0.001	0.664	6	8.182	<0.001	0.577	3	10.649	<0.001	0.470	2	97.969	<0.001	0.942	1	25.723	<0.001	0.682	3	29.319	<0.001	0.710
**Y(NA)**	6	15.322	<0.001	0.719	2	24.440	<0.001	0.803	6	9.584	<0.001	0.615	3	22.667	<0.001	0.654	2	34.506	<0.001	0.852	1	0.095	0.763	0.008	3	70.311	<0.001	0.854
**Chl *a***	6	1.763	0.135	0.227	2	16.976	<0.001	0.739	6	8.977	<0.001	0.599	3	3.993	0.015	0.250	2	41.294	<0.001	0.873	1	160.007	<0.001	0.930	3	23.589	<0.001	0.663
**Chl *c***	6	1.468	0.217	0.197	2	16.423	<0.001	0.732	6	5.227	0.001	0.466	3	14.096	<0.001	0.540	2	60.837	<0.001	0.910	1	36.778	<0.001	0.754	3	27.041	<0.001	0.693
**Fux**	6	3.590	0.007	0.374	2	3.288	0.073	0.354	6	0.763	0.603	0.113	3	8.185	<0.001	0.405	2	33.722	<0.001	0.849	1	102.146	<0.001	0.895	3	44.945	<0.001	0.789
**Pro**	6	7.052	<0.001	0.540	2	3.254	0.074	0.352	6	88.485	<0.001	0.936	3	41.300	<0.001	0.775	2	259.158	<0.001	0.977	1	162.950	<0.001	0.931	3	247.639	<0.001	0.954
**MDA**	6	4.183	0.003	0.411	2	4.575	0.033	0.433	6	36.176	<0.001	0.858	3	10.371	<0.001	0.464	2	289.777	<0.001	0.980	1	129.892	<0.001	0.915	3	206.871	<0.001	0.945
**TAC**	6	3.051	0.016	0.337	2	148.393	<0.001	0.961	6	28.969	<0.001	0.828	3	27.169	<0.001	0.694	2	281.955	<0.001	0.979	1	15.734	0.002	0.567	3	89.866	<0.001	0.882
**SOD**	6	7.086	<0.001	0.541	2	207.056	<0.001	0.972	6	16.022	<0.001	0.728	3	79.716	<0.001	0.869	2	718.147	<0.001	0.992	1	3.857	0.073	0.243	3	66.812	<0.001	0.848
**CAT**	6	8.007	<0.001	0.572	2	375.889	<0.001	0.984	6	30.284	<0.001	0.835	3	8.296	<0.001	0.409	2	904.183	<0.001	0.993	1	22.869	<0.001	0.656	3	175.576	<0.001	0.936
**POD**	6	6.941	<0.001	0.536	2	385.287	<0.001	0.985	6	0.652	0.688	0.098	3	26.629	<0.001	0.689	2	319.478	<0.001	0.982	1	31.559	<0.001	0.725	3	47.611	<0.001	0.799

Table S3. Simple effects of marine heatwave intensity on physiological and biochemical indicators of *Undaria pinnatifida*, with light intensity and sampling time held constant.

Factors			***P* Value**											
**Light**	**Time**	**MHW**	**RGR**	**F_v/F_m**	**Y(II)**	**Y(NPQ)**	**Y(NO)**	**Fux**	**Pro**	**MDA**	**TAC**	**SOD**	**CAT**	**POD**
Normal	WP	Moderate - Severe	0.033	0.110	0.159	0.554	1.000	0.061	0.016	1.000	0.024	0.281	0.002	0.001
		Moderate - Control	1.000	0.003	1.000	1.000	0.731	0.488	0.005	<0.001	<0.001	<0.001	<0.001	<0.001
		Severe - Control	0.063	0.222	0.232	0.124	1.000	0.778	<0.001	<0.001	<0.001	<0.001	<0.001	0.001
	PP	Moderate - Severe	0.054	<0.001	0.182	1.000	0.052	0.096	0.037	0.025	0.148	0.048	0.175	0.013
		Moderate - Control	0.590	1.000	0.006	1.000	<0.001	0.026	0.404	0.022	<0.001	<0.001	<0.001	<0.001
		Severe - Control	0.588	<0.001	<0.001	1.000	<0.001	1.000	0.002	<0.001	<0.001	<0.001	<0.001	<0.001
	CP	Moderate - Severe	<0.001	0.241	<0.001	0.001	0.001	0.069	<0.001	1.000	<0.001	0.007	0.159	1.000
		Moderate - Control	1.000	0.003	<0.001	<0.001	<0.001	1.000	0.006	<0.001	<0.001	<0.001	<0.001	<0.001
		Severe - Control	<0.001	<0.001	<0.001	<0.001	<0.001	0.143	<0.001	<0.001	<0.001	<0.001	<0.001	<0.001
	RP	Moderate - Severe	<0.001	0.386	0.346	0.001	0.008	0.011	<0.001	<0.001	0.001	1.000	<0.001	0.078
		Moderate - Control	1.000	0.002	<0.001	0.411	<0.001	0.479	0.513	<0.001	<0.001	<0.001	<0.001	<0.001
		Severe - Control	<0.001	<0.001	<0.001	0.023	<0.001	0.001	<0.001	<0.001	<0.001	<0.001	<0.001	<0.001
High	WP	Moderate - Severe	<0.001	0.193	0.138	0.848	0.017	0.004	<0.001	0.714	0.874	0.078	<0.001	1.000
		Moderate - Control	0.748	0.001	0.009	1.000	0.024	0.124	0.011	<0.001	0.017	0.001	0.001	1.000
		Severe - Control	0.004	<0.001	0.472	0.557	1.000	0.279	<0.001	<0.001	0.002	<0.001	1.000	1.000
	PP	Moderate - Severe	<0.001	<0.001	1.000	<0.001	<0.001	0.145	0.143	<0.001	<0.001	<0.001	0.020	0.001
		Moderate - Control	0.199	1.000	0.010	<0.001	0.060	0.375	1.000	1.000	0.001	<0.001	0.001	1.000
		Severe - Control	0.001	<0.001	0.053	0.363	<0.001	1.000	0.055	<0.001	1.000	0.001	0.206	<0.001
	CP	Moderate - Severe	<0.001	<0.001	<0.001	<0.001	<0.001	0.001	0.054	0.983	<0.001	0.001	<0.001	0.001
		Moderate - Control	0.002	0.271	<0.001	<0.001	0.074	0.003	<0.001	<0.001	0.004	<0.001	<0.001	1.000
		Severe - Control	0.275	<0.001	<0.001	<0.001	<0.001	1.000	<0.001	<0.001	0.165	0.453	0.018	0.003
	RP	Moderate - Severe	<0.001	0.022	1.000	<0.001	<0.001	<0.001	<0.001	<0.001	<0.001	<0.001	<0.001	0.142
		Moderate - Control	<0.001	0.004	0.008	<0.001	<0.001	<0.001	0.039	0.130	<0.001	<0.001	<0.001	1.000
		Severe - Control	0.001	1.000	0.018	0.005	1.000	1.000	<0.001	<0.001	<0.001	0.394	0.005	0.096

Table S4. Simple effects of light intensity on physiological and biochemical indicators of *Undaria pinnatifida*, with marine heatwave intensity and sampling time held constant.

Factors			*P* Value											
MHW	Time	Light	RGR	F_v/F_m	Y(II)	Y(NPQ)	Y(NO)	Fux	Pro	MDA	TAC	SOD	CAT	POD
Control	WP	Normal - High	0.248	0.525	0.358	0.832	0.536	0.687	0.776	0.029	<0.001	<0.001	<0.001	<0.001
	PP	Normal - High	0.164	0.010	<0.001	<0.001	0.154	0.002	0.001	0.011	<0.001	<0.001	<0.001	<0.001
	CP	Normal - High	<0.001	<0.001	0.269	0.350	0.877	0.007	<0.001	<0.001	<0.001	<0.001	<0.001	<0.001
	RP	Normal - High	<0.001	<0.001	0.065	<0.001	<0.001	0.281	<0.001	<0.001	<0.001	0.003	<0.001	<0.001
Moderate	WP	Normal - High	0.730	0.760	<0.001	0.709	<0.001	0.089	0.501	0.055	0.035	0.457	0.096	0.205
	PP	Normal - High	0.054	0.016	<0.001	0.886	<0.001	0.002	0.010	0.445	0.085	0.094	0.684	<0.001
	CP	Normal - High	0.051	<0.001	<0.001	0.884	<0.001	<0.001	<0.001	<0.001	<0.001	0.006	<0.001	0.020
	RP	Normal - High	<0.001	<0.001	<0.001	0.969	<0.001	0.011	<0.001	0.126	<0.001	<0.001	<0.001	<0.001
Severe	WP	Normal - High	0.017	0.001	0.604	0.519	0.228	0.250	0.061	0.029	0.001	0.961	0.014	0.005
	PP	Normal - High	0.036	0.039	0.000	<0.001	0.205	0.024	0.038	0.001	<0.001	<0.001	<0.001	<0.001
	CP	Normal - High	0.141	<0.001	<0.001	<0.001	0.001	<0.001	0.001	0.006	<0.001	0.001	<0.001	<0.001
	RP	Normal - High	0.005	<0.001	0.001	0.192	0.002	<0.001	0.001	0.040	<0.001	<0.001	0.031	<0.001

Table S5. Simple effects of sampling time on physiological and biochemical indicators of *Undaria pinnatifida*, with marine heatwave intensity and light intensity held constant.

Factors			***P*** **Value**											
**MHW**	**Light**	**Time**	**RGR**	**F_v/F_m**	**Y(II)**	**Y(NPQ)**	**Y(NO)**	**Fux**	**Pro**	**MDA**	**TAC**	**SOD**	**CAT**	**POD**
Control	Normal	WP-PP	1.000	1.000	0.003	1.000	0.003	1.000	1.000	1.000	0.508	1.000	1.000	1.000
		WP-CP	1.000	1.000	<0.001	0.000	<0.001	0.137	1.000	1.000	1.000	0.862	1.000	1.000
		WP-RP	1.000	1.000	<0.001	0.263	<0.001	0.005	1.000	1.000	0.182	1.000	1.000	1.000
		PP-CP	1.000	1.000	<0.001	<0.001	<0.001	0.034	1.000	1.000	1.000	1.000	1.000	1.000
		PP-RP	0.407	1.000	<0.001	0.207	0.001	0.004	1.000	1.000	1.000	1.000	1.000	1.000
		CP-RP	1.000	1.000	0.002	0.002	1.000	0.020	1.000	0.937	0.373	1.000	1.000	1.000
	High	WP-PP	1.000	0.005	<0.001	0.001	0.120	0.074	0.008	0.222	0.226	<0.001	1.000	1.000
		WP-CP	<0.001	<0.001	<0.001	<0.001	<0.001	0.001	<0.001	<0.001	0.001	<0.001	0.007	1.000
		WP-RP	<0.001	<0.001	<0.001	<0.001	1.000	0.002	<0.001	<0.001	0.000	<0.001	0.000	0.001
		PP-CP	<0.001	<0.001	<0.001	0.023	<0.001	1.000	1.000	0.028	0.164	1.000	0.001	0.037
		PP-RP	<0.001	<0.001	<0.001	<0.001	0.517	1.000	<0.001	0.003	0.014	<0.001	<0.001	<0.001
		CP-RP	0.098	0.018	0.036	0.003	<0.001	0.917	<0.001	1.000	1.000	0.010	0.030	<0.001
Moderate	Normal	WP-PP	0.589	0.005	0.643	1.000	0.395	1.000	1.000	1.000	1.000	0.039	1.000	1.000
		WP-CP	1.000	0.355	0.529	1.000	0.484	0.395	0.804	<0.001	1.000	0.814	1.000	1.000
		WP-RP	1.000	0.123	0.225	1.000	0.069	0.007	1.000	<0.001	1.000	0.388	0.001	0.332
		PP-CP	0.040	0.001	1.000	1.000	1.000	0.127	0.357	<0.001	1.000	1.000	0.437	0.014
		PP-RP	0.005	0.001	1.000	1.000	1.000	0.006	1.000	0.001	1.000	1.000	0.008	0.021
		CP-RP	1.000	1.000	1.000	1.000	1.000	0.003	0.073	0.586	0.501	1.000	0.073	1.000
	High	WP-PP	0.051	0.930	1.000	1.000	1.000	0.763	0.050	1.000	0.998	0.347	0.718	1.000
		WP-CP	0.047	<0.001	0.013	1.000	0.054	0.001	<0.001	<0.001	0.003	0.021	0.040	0.892
		WP-RP	0.003	<0.001	0.002	1.000	0.001	0.004	<0.001	<0.001	0.004	0.001	<0.001	<0.001
		PP-CP	<0.001	<0.001	0.030	1.000	0.014	0.099	<0.001	<0.001	0.006	0.013	0.001	1.000
		PP-RP	<0.001	<0.001	0.010	1.000	0.004	0.518	0.160	<0.001	0.001	<0.001	<0.001	<0.001
		CP-RP	1.000	1.000	1.000	1.000	1.000	0.390	<0.001	0.002	0.872	0.559	0.027	<0.001
Severe	Normal	WP-PP	0.017	<0.001	0.343	0.884	1.000	1.000	1.000	0.008	0.001	0.421	<0.001	0.005
		WP-CP	<0.001	<0.001	<0.001	1.000	0.002	1.000	<0.001	<0.001	<0.001	<0.001	0.263	0.008
		WP-RP	<0.001	<0.001	0.191	0.039	0.742	1.000	<0.001	<0.001	<0.001	1.000	<0.001	0.768
		PP-CP	<0.001	1.000	<0.001	0.047	<0.001	1.000	<0.001	0.003	0.007	0.019	<0.001	1.000
		PP-RP	<0.001	1.000	0.005	<0.001	1.000	1.000	<0.001	<0.001	0.123	0.083	<0.001	0.065
		CP-RP	0.018	1.000	0.015	0.096	<0.001	1.000	<0.001	0.009	0.003	0.003	<0.001	0.084
	High	WP-PP	<0.001	0.002	0.013	0.004	1.000	0.131	0.057	<0.001	<0.001	<0.001	0.024	0.013
		WP-CP	<0.001	<0.001	<0.001	<0.001	1.000	<0.001	<0.001	<0.001	<0.001	<0.001	1.000	0.002
		WP-RP	<0.001	<0.001	<0.001	0.001	1.000	<0.001	<0.001	<0.001	<0.001	<0.001	<0.001	<0.001
		PP-CP	<0.001	<0.001	<0.001	<0.001	0.414	0.063	<0.001	0.009	0.012	0.052	0.001	0.578
		PP-RP	<0.001	0.001	0.015	0.327	0.250	0.053	<0.001	<0.001	<0.001	<0.001	<0.001	0.001
		CP-RP	0.006	0.012	0.084	0.019	1.000	1.000	<0.001	0.035	0.003	0.007	<0.001	0.004

Table S6. Simple effects of marine heatwave intensity at each light level on physiological and biochemical indicators of *Undaria pinnatifida*.

Factors		*P* Value		
**Light**	**MHW**	**qP**	**Chl *a***	**Chl *c***
Normal	Moderate - Severe	<0.001	1.000	<0.001
	Moderate - Control	1.000	<0.001	<0.001
	Severe - Control	<0.001	<0.001	<0.001
High	Moderate - Severe	0.305	0.005	0.007
	Moderate - Control	0.979	1.000	1.000
	Severe - Control	0.048	0.004	0.003

Table S7. Data quality of *Undaria pinnatifida* transcriptome under different marine heatwave and light treatments.

Sample	Raw reads	Raw bases	Clean reads	Clean bases	Error rate (%)	Q20 (%)	Q30 (%)	GC ratio (%)
Ctrl1	23561891	7.1	22788526	6.8	0.01	98.21	94.96	55.12
Ctrl2	22533001	6.8	21954375	6.6	0.01	97.95	94.29	54.82
Ctrl3	22673071	6.8	21749041	6.5	0.01	98.14	94.82	54.81
$H_{Light}1$	23482533	7	22742535	6.8	0.01	97.96	94.36	54.33
$H_{Light}2$	21073750	6.3	20508302	6.2	0.01	97.91	94.22	53.59
$H_{Light}3$	21437231	6.4	20737272	6.2	0.01	97.5	93.17	53.75
$M_{MHW}N_{Light}1$	23211683	7	21916593	6.6	0.01	98.02	94.46	54.82
$M_{MHW}N_{Light}2$	23339700	7	22692577	6.8	0.01	97.97	94.35	54.67
$M_{MHW}N_{Light}3$	21882524	6.6	21213742	6.4	0.01	98.08	94.57	54.6
$M_{MHW}H_{Light}1$	22712264	6.8	22075514	6.6	0.01	98.03	94.47	54.93
$M_{MHW}H_{Light}2$	22739221	6.8	22141681	6.6	0.01	98.18	94.9	54.83
$M_{MHW}H_{Light}3$	20105617	6	19546567	5.9	0.01	98.01	94.4	54.86
$S_{MHW}N_{Light}1$	23961216	7.2	23347825	7	0.01	98.18	94.92	53.52
$S_{MHW}N_{Light}2$	21621916	6.5	21038470	6.3	0.01	97.96	94.3	53.63
$S_{MHW}N_{Light}3$	23816505	7.1	23127512	6.9	0.01	98.05	94.47	54.15
$S_{MHW}H_{Light}1$	23319138	7	22734451	6.8	0.01	98.04	94.5	53.39
$S_{MHW}H_{Light}2$	20269493	6.1	19757513	5.9	0.01	98.04	94.49	54.1
$S_{MHW}H_{Light}3$	22640174	6.8	22040765	6.6	0.01	98.02	94.42	53.48

Table S8. Single-gene functional annotation of *Undaria pinnatifida* transcriptome in seven databases under different marine heatwave and light treatments.

Gene Annotation Databases	Number of Unigenes	Percentage (%)
Annotated in NR	13671	8.16
Annotated in NT	1917	1.14
Annotated in KO	7698	4.59
Annotated in SwissProt	12210	7.29
Annotated in PFAM	40379	24.12
Annotated in GO	40379	24.12
Annotated in KOG	7089	4.23
Annotated in all Databases	901	0.53
Annotated in at least one Database	44859	26.8
Total Unigenes	167374	100
